# Complex nutrient channel phenotypes despite Mendelian inheritance in a *Plasmodium falciparum* genetic cross

**DOI:** 10.1371/journal.ppat.1008363

**Published:** 2020-02-18

**Authors:** Ankit Gupta, Abdullah A. B. Bokhari, Ajay D. Pillai, Anna K. Crater, Jeanine Gezelle, Gagandeep Saggu, Armiyaw S. Nasamu, Suresh M. Ganesan, Jacquin C. Niles, Sanjay A. Desai

**Affiliations:** 1 Laboratory of Malaria and Vector Research, National Institute of Allergy and Infectious Diseases, National Institutes of Health, Rockville, Maryland, United States of America; 2 Department of Biological Engineering, Massachusetts Institute of Technology, Cambridge, Massachusetts, United States of America; 3 Division of Comparative Medicine, Massachusetts Institute of Technology, Cambridge, Massachusetts, United States of America; Seattle Children's Research Institute, UNITED STATES

## Abstract

Malaria parasites activate a broad-selectivity ion channel on their host erythrocyte membrane to obtain essential nutrients from the bloodstream. This conserved channel, known as the plasmodial surface anion channel (PSAC), has been linked to parasite *clag3* genes in *P*. *falciparum*, but epigenetic switching between the two copies of this gene hinders clear understanding of how the encoded protein determines PSAC activity. Here, we used linkage analysis in a *P*. *falciparum* cross where one parent carries a single *clag3* gene to overcome the effects of switching and confirm a primary role of the *clag3* product with high confidence. Despite Mendelian inheritance, CLAG3 conditional knockdown revealed remarkably preserved nutrient and solute uptake. Even more surprisingly, transport remained sensitive to a CLAG3 isoform-specific inhibitor despite quantitative knockdown, indicating that low doses of the CLAG3 transgene are sufficient to confer block. We then produced a complete CLAG3 knockout line and found it exhibits an incomplete loss of transport activity, in contrast to *rhoph2* and *rhoph3*, two PSAC-associated genes that cannot be disrupted because nutrient uptake is abolished in their absence. Although the CLAG3 knockout did not incur a fitness cost under standard nutrient-rich culture conditions, this parasite could not be propagated in a modified medium that more closely resembles human plasma. These studies implicate oligomerization of CLAG paralogs encoded by various chromosomes in channel formation. They also reveal that CLAG3 is dispensable under standard *in vitro* conditions but required for propagation under physiological conditions.

## Author summary

Malaria, a globally important infectious disease, is caused by parasites that invade and grow in circulating red blood cells to avoid host immune attack. Infected red blood cells have increased uptake of diverse nutrients, fueling parasite growth; this uptake is mediated by an ion channel that transports essential nutrients across the red blood cell membrane. Three proteins made by the parasite have been linked to this channel, but how they increase uptake is unknown. Here, we used mapping in a genetic cross of two strains of the virulent human malaria parasite to confirm a primary role of one protein known as CLAG3. We then used gene editing to produce a parasite that has reduced CLAG3 levels when a stabilizing chemical is removed; surprisingly, solute transport was minimally changed despite a 90% reduction in CLAG3. Gene editing was also used to make a parasite without any CLAG3. This knockout parasite had reduced nutrient uptake, but it still grew normally in media with high nutrient levels; it was unable to grow when nutrient levels were lowered to levels like those in the human bloodstream. The complex effects of channel inhibitors on these genetically modified parasites suggests that CLAG3 and the two other proteins interact with each other to form large protein clusters in the red blood cell membrane; these clusters may form the nutrient uptake pore. Our studies indicate that CLAG3 is required for parasite survival and growth in the bloodstream and that the channel it produces can be targeted to make new antimalarial drugs.

## Introduction

Malaria remains an important global health concern as effective vaccines are unavailable and acquired resistance to approved drugs may undermine progress made in the last two decades [1]. As much of the recent progress is attributed to mosquito control [2], increasing rates of insecticide resistance in these disease vectors is also worrisome for achieving the goal of malaria eradication [3]. There is therefore a concerted effort to identify new drug and vaccine targets [4].

Most of the symptoms and sequalae of malaria result from parasite growth and replication within circulating erythrocytes in the animal host. The virulent human parasite, *P*. *falciparum*, significantly remodels its host cell by exporting hundreds of effector proteins into erythrocyte cytosol [5,6]. These proteins establish membranous structures in the host cell [7], alter erythrocyte deformability [8], mediate cytoadherence and immune evasion [9,10], and facilitate uptake of nutrients and essential ions [11].

An unusual, small conductance ion channel known as the plasmodial surface anion channel (PSAC) is now established as the primary uptake mechanism for a broad range of nutrients, anions, and organic cations [12], but does not account for an increased Ca^++^ permeability of infected cells [13]. Target-based chemical screens have identified potent PSAC inhibitors that sterilize parasite cultures and are being pursued for antimalarial drug development [14]. Interestingly, while PSAC inhibitors have relatively poor efficacies against *in vitro* parasite growth when examined using standard RPMI 1640 media containing supraphysiological nutrient concentrations, they kill parasites at low nanomolar concentrations if tested with a modified medium that has nutrient levels closer to those in human plasma [14]. These findings have established that PSAC serves an essential role in parasite nutrient acquisition and stimulated antimalarial drug development against this target.

PSAC inhibitors have also proven to be important tools for identifying the parasite genes responsible for this channel. Inhibitor screens identified an isolate-specific PSAC antagonist, ISPA-28, that selectively blocks channels associated with the Dd2 parasite line, but not those from unrelated *P*. *falciparum* lines [12]. Genetic mapping of channel block in a Dd2 x HB3 genetic cross and DNA transfection experiments implicated two *clag3* genes from chromosome 3 in the parasite genome. Consistent with a role in PSAC formation, the encoded CLAG3 protein localizes to the host membrane with a variant surface-exposed motif required for ISPA-28 binding [15]. CLAG3 and its paralogs from other chromosomes traffic to the host membrane and appear to be the only conserved surface-exposed antigens on primate, rodent, and avian erythrocytes infected with their respective *Plasmodium spp*. [16–18], paralleling functional conservation of PSAC activity in malaria parasites [19]. RhopH2 and RhopH3, unrelated proteins that associate with CLAG proteins to form the RhopH complex, are also strictly conserved in *Plasmodium spp*. and have been linked to PSAC activity [20–22]. Finally, additional independent support for CLAG3 involvement has come from *in vitro* selection of functional mutants carrying either a CLAG3 mutation or near-complete silencing of *clag3* and a chromosome 2 paralog termed *clag2* [23–25].

Despite these independent lines of evidence, there are still many unknowns about these proteins and their contributions to PSAC activity. None of the identified proteins (CLAGs, RhopH2, and RhopH3) have detectable homology to known ion channels; even when considered together, they appear to lack the number of transmembrane domains required to form a stable aqueous pore [26]. The roles of *clag* paralogs on other chromosomes—*clag2*, *clag8*, and *clag9*—in the *P*. *falciparum* genome are largely unexplored; expansion of this multigene family appears to be ongoing [27]. It is also unclear why *clag* genes show variable expansion in other *Plasmodia*, with some species having up to 35 copies [28,29]. The two *clag3* genes in *P*. *falciparum* undergo epigenetic switching [12,30,31], but the benefit to the parasite is unclear. Although parasite multigene families typically use gene switching for immune evasion [32], an interesting alternative proposal is that switching between the two *clag3* genes alters channel properties and enables optimal nutrient uptake that can respond to the human host’s nutritional status [33]. Because these proteins are broadly acknowledged drug and vaccine targets [14,21,34,35], these and various other questions about the RhopH complex and the roles of the member proteins in intracellular parasite development will require concerted study.

Here, we examined CLAG3 requirement and functional roles in parasites that have either one or two copies of the *clag3* gene. We began with linkage analysis for inheritance of differential PSAC block by a novel inhibitor in the GB4 x 7G8 *P*. *falciparum* genetic cross [36], where GB4 and many progeny clones have only one *clag3* gene instead of the two copies present in most field and laboratory lines. This copy number reduction permitted high confidence genetic mapping of the *clag3* locus, with a logarithm of odds (LOD) score exceeding those previously reported for any *P*. *falciparum* phenotype. We then used DNA transfection to produce *clag3* conditional knockdown and knockout lines, uncovering the complex contributions of these proteins in channel formation and nutrient acquisition. These studies provide additional validation of a conserved, essential antimalarial drug target and clarify possible models for PSAC composition, structure and function.

## Results

### Copy number reduction permits high confidence genetic mapping of CLAG3 in PSAC inhibitor phenotype

**Fig 1:**
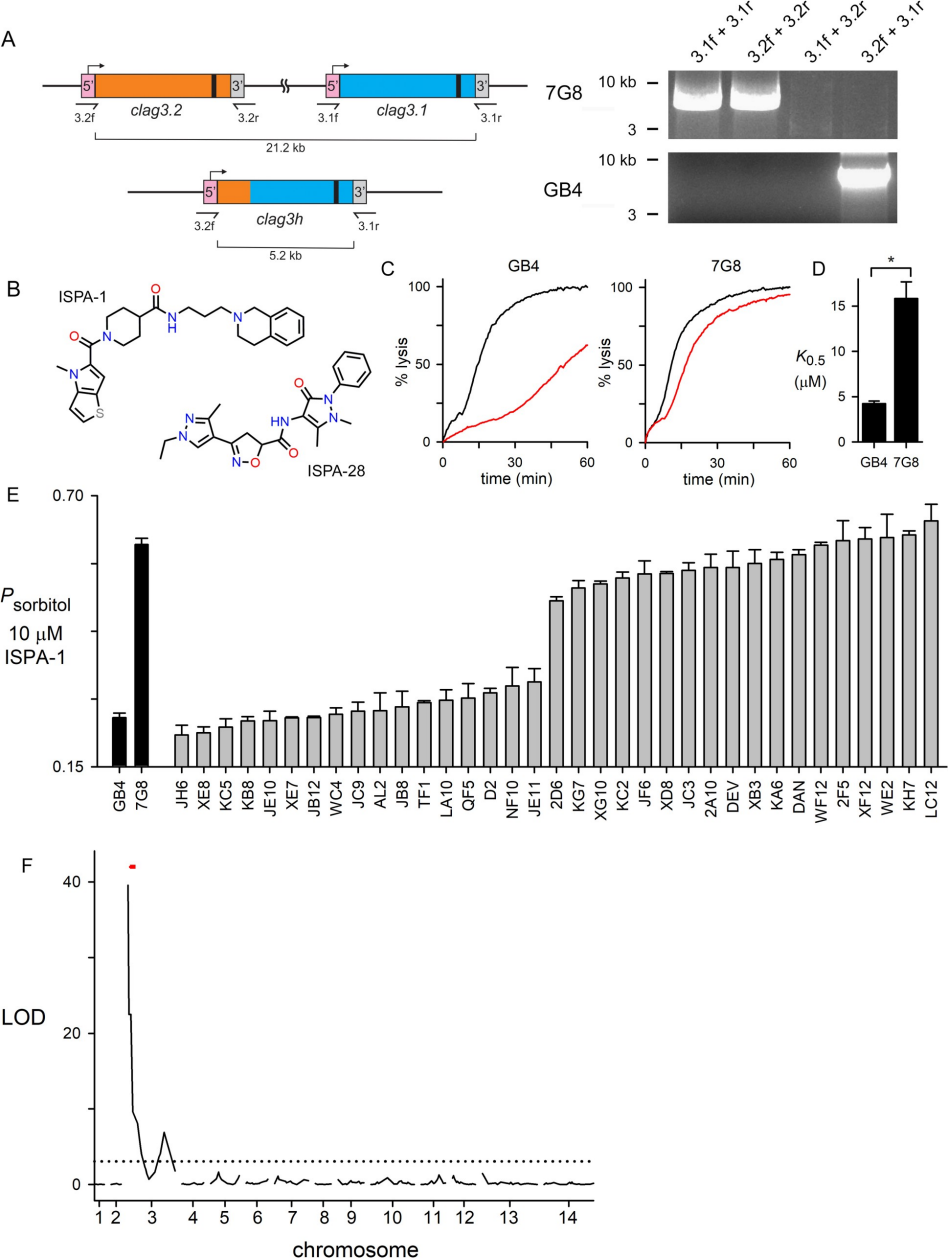
Mendelian inheritance of a PSAC phenotype in the GB4 x 7G8 cross. (A) Schematic showing the two *clag3* genes in 7G8 and a single *clag3h* in GB4; gene ribbons are color-coded to show site of recombination to produce copy number reduction in GB4. Black bar near the 3’ end of each gene represents a variant region that encodes a CLAG3 extracellular loop implicated in binding of isolate-specific inhibitors. Primer binding sites are indicated. Ethidium-stained gel at right shows PCR confirming the differences in *clag3* copy number. (B) Structures of ISPA-1 and ISPA-28. (C) Sorbitol-induced osmotic lysis kinetics for GB4 and 7G8 without and with 10 μM ISPA-1 (black and red traces in each panel, respectively). ISPA-1 produces greater block of GB4 channels, as indicated by slower sorbitol-induced lysis. (D) Mean ± S.E.M. half-maximal inhibitory concentrations, *K*_*0*.*5*_, for ISPA-1, determined from dose response experiments as in panel C. *, *P* < 10^−4^. (E) Mean ± S.E.M. sorbitol permeability with 10 μM ISPA-1, normalized to 1.0 for matched trials without ISPA-1. Results are shown for indicated parental lines and progeny clones from the genetic cross (*n* = 13–16 and 2–5 for parental and progeny clones, respectively). Each progeny’s phenotype matches that of a parent. (F) Logarithm of odds (LOD) scores from a primary scan of QTL for ISPA-1 affinity. The peak (LOD of 40) maps to the 5’ end of chromosome 3 and includes the *clag3* locus (red dash). Dashed line indicates the *P* = 0.05 significance threshold, calculated from 1000 permutations.

While most field and laboratory lines of *P*. *falciparum* have two *clag3* genes referred to as *clag3*.*1* and *clag3*.*2*, some parasite lines have undergone copy number reduction through homologous recombination between the two paralogs located 16 kB apart on chromosome 3 [34]. As the single *clag3* gene in these lines carries the *5’* UTR of *clag3*.*2* and the *3’* UTR of *clag3*.*1*, this hybrid gene is termed *clag3h*. We used primers specific to the UTRs of each paralog to determine that GB4 and 7G8, the parents of a *P*. *falciparum* genetic cross [36], carry one and two copies, respectively (Fig 1A). The proteins encoded by these *clag3* alleles are more than 90% identical, with most of the variation present in a small hypervariable region near the C-terminus (HVR, S1A Fig). The unique HVR sequence of the Dd2 CLAG3.1 protein is responsible for PSAC block by ISPA-28 in this laboratory clone and lack of activity against channels from other parasite lines [15,37]. In keeping with this, we found that ISPA-28 is ineffective against 7G8 and GB4 channels (Fig 1B and S1B Fig). While ISPA-28 was instrumental in mapping studies using the Dd2 x HB3 to implicate CLAG3 [12], this compound does not produce heritable differences in PSAC phenotype between the 7G8 and GB4 lines and is, therefore, not useful for linkage studies in their genetic cross.

Because reduced *clag3* copy number in GB4 should be inherited in a subset of progeny, we reasoned that the GB4 x 7G8 cross may provide unique insights into the role of the *clag3* product. We therefore surveyed hits from previous PSAC inhibitor screens to identify ISPA-1, a relatively low affinity inhibitor that blocks GB4 channels with 4-fold greater affinity than those associated with 7G8 parasites (Fig 1B–1D). Using a kinetic assay that continuously tracks osmotic lysis resulting from PSAC-mediated sorbitol uptake, the *K*_*0*.*5*_ values for channel block were 4.2 ± 0.3 and 15.8 ± 1.8 μM for GB4 and 7G8 parasites, respectively (*P* < 10^-4^, *n* = 5 dose response experiments each). We then examined inheritance of PSAC block in the cross progeny using a 10 μM ISPA-1 concentration in osmotic lysis experiments and found that all 35 progeny clones matched one or the other parental line (Fig 1E), suggesting monogenic inheritance.

We then used quantitative trait locus (QTL) mapping to identify possible parasite genomic loci that define ISPA-1 block of channels in this cross (Fig 1F). A primary scan using defined microsatellite markers and single nucleotide polymorphisms revealed a single locus at the 5’ end of chromosome 3 with a logarithm of odds (LOD) score of 40.0, exceeding previously reported scores for examined *P*. *falciparum* phenotypes. The mapped locus of 139.5 kB contains 32 annotated genes (S1 Table) including two *clag3* genes mapped by previous linkage analysis studies of PSAC activity in other genetic crosses [12,14,38]. This result provides independent evidence for this locus in solute and nutrient trafficking into infected erythrocytes.

Because the progeny clones in Fig 1E segregated into two groups of essentially equal size, these data also argue against a survival advantage of either parental allele at the responsible locus during *in vivo* expansion in chimpanzees, as required to produce this genetic cross; absence of preferential inheritance also argues against epistatic interactions between this locus and other parasite genomic loci [39].

### Conditional CLAG3 knockdown reveals an unexpected dose effect on PSAC activity and inhibitor efficacy

Because CLAG3 plays a pivotal role in solute transport and parasite propagation, we used DNA transfection to produce parasite lines with conditional reductions in CLAG3 protein. Prior studies have reported reduced growth phenotypes with CLAG3 knockdown achieved through either epigenetic silencing or *in vitro* selection [24,25,40], but none have clearly linked loss of CLAG3 to compromised parasite survival. Because exported proteins such as CLAG3 may be less effectively knocked down by conventional translation-level repression methods [41], we selected the recently developed TetR-DOZI system for conditional expression [42]. This system utilizes an RNA-aptamer sequence in the 3’ UTR of a target gene’s mRNA to recruit a fusion protein consisting of the Tet repressor protein (TetR) and DOZI (development of zygote inhibited), a DDX6 helicase protein that represses translation [43]. This two-component fusion improves the effectiveness of knockdown and is suitable for targeting both soluble and membrane-associated proteins in *P*. *falciparum* [42,44].

**Fig 2:**
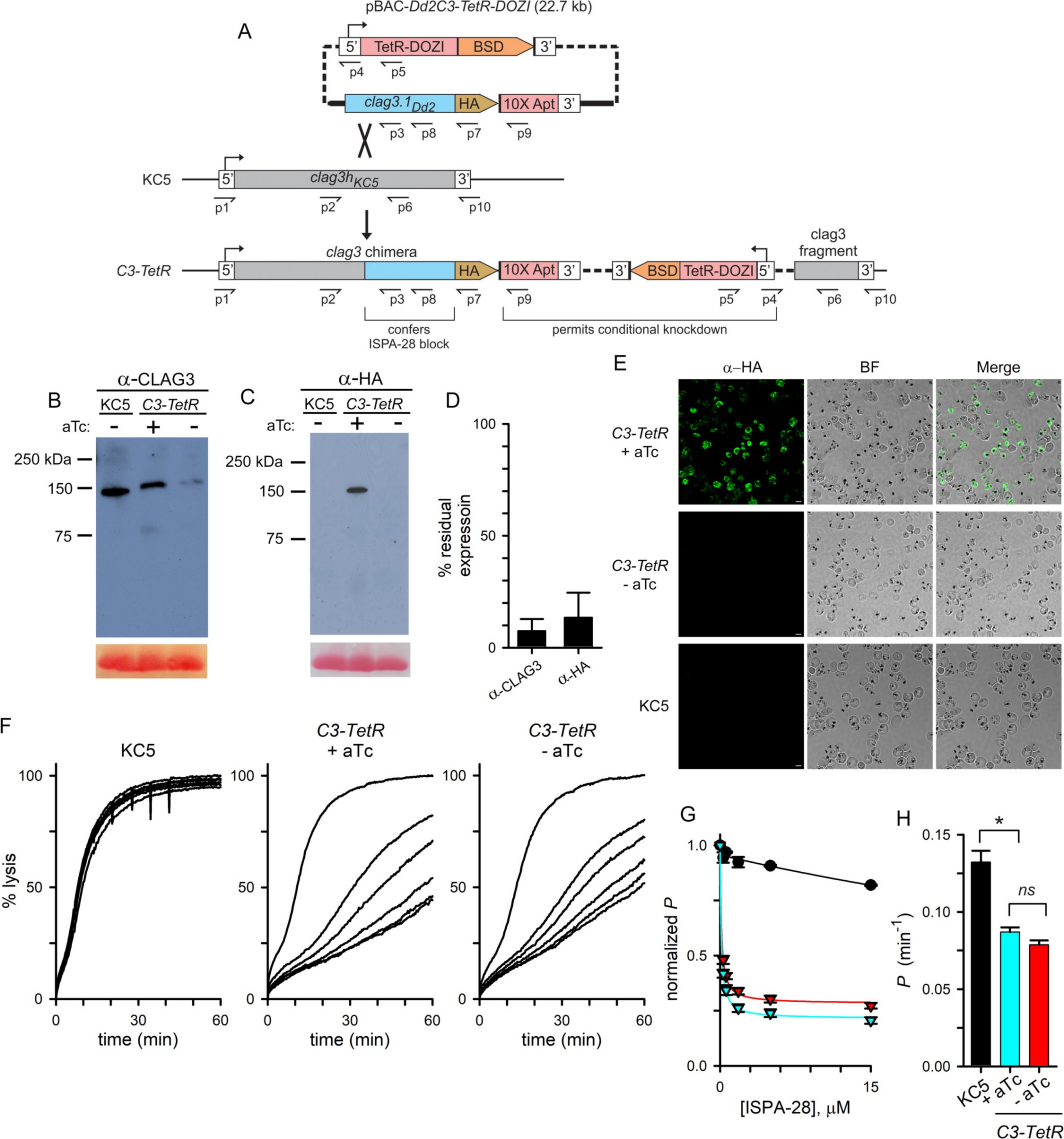
CLAG3 conditional knockdown reveals complex effects on channel pharmacology and transport rates. (A) Allelic exchange strategy for production of a chimeric *clag3* gene and conditional knockdown. The TetR-DOZI conditional regulator and blasticidin S deaminase (BSD) are expressed under a single promoter and separated by a viral 2A skip peptide [75, not illustrated]. A C-terminal HA epitope tag (HA) is appended to the *clag3* gene and is followed by a stop codon and a 10x aptamer sequence (10X Apt) embedded in the 3’ UTR. Primer positions are shown as used for PCR results in S2 Fig; primer sequences are listed in S2 Table. (B-C) Immunoblots using total cell lysates from indicated parasites cultivated with or without 2 μM aTc, probed with CLAG3- or HA epitope tag-specific antibody (panels B and C, respectively). Bottom shows Ponceau S staining of hemoglobin as a loading control. (D) Digital quantification of residual CLAG3 expression upon aTc removal to produce knockdown. Mean ± S.E.M. band densities from 3 independent harvests and immunoblots as in panels B and C. (E) Indirect immunofluorescence images of trophozoite-stage *C3-TetR* and KC5 wildtype parasites probed with anti-HA antibody (green). *C3-TetR* parasites are shown after cultivation with or without aTc. Scale bar, 5 μm. (F) Continuous recordings of sorbitol-induced osmotic lysis kinetics for the parental KC5 line or the *C3-TetR* line cultivated with or without aTc, as indicated. Traces reflect lysis kinetics with 0, 0.3, 0.6, 1.8, 5, or 15μM ISPA-28 (top to bottom, respectively in each panel). (G) Dose responses for ISPA-28 inhibition for KC5 (black circles) and *C3-TetR* grown with or without aTc (blue and red triangles, respectively). Symbols represent mean ± S.E.M. permeabilities, normalized to 1.0 for matched traces without inhibitor; *n* = 5–7 trials at each concentration. Solid lines represent best fits to *y = a*/(1 + (*x*/*b*)) + (1-*a*)/(1 + (*x*/*c*)). (H) Mean ± S.E.M. apparent sorbitol permeabilities for indicated lines, calculated as the reciprocal of the inhibitor-free lysis halftime; *n* = 5–7 trials each. *, *p* < 10^−4^; *ns*, not significantly different.

We designed and produced a single plasmid strategy for allelic exchange recombination to achieve efficient CLAG3 knockdown (Fig 2A). The *pBAC-Dd2C3-TetR-DOZI* plasmid carries a 3.2 kB fragment from the end of the Dd2 *clag3*.*1* gene with an in-frame C-terminal HA epitope tag and a 10x aptamer sequence in the 3’UTR for recruitment of TetR-DOZI. We selected KC5, a progeny clone from the GB4 x 7G8 cross, for transfection because it carries a single *clag3h* gene and has been successfully used in prior studies [37]; a single *clag3h* gene avoids epigenetic switching between the desired integrated gene and a second unmodified *clag3* gene [12].

We anticipated homologous recombination between the KC5 *clag3h* genomic site and the Dd2 *clag3*.*1* gene fragment on the plasmid as these *clag3* genes are nearly identical. This recombination adds a C-terminal HA epitope tag to the encoded protein and, importantly, confers PSAC block by ISPA-28, an inhibitor specific for Dd2 *clag3*.1-associated channels; ISPA-28 sensitivity has been mapped to a variant motif near the C-terminus of the encoded protein [15]. As the transfection plasmid carries only a *clag3* fragment without a promoter sequence, changes in PSAC phenotype can only be observed after in-frame integration into the genomic *clag3h*. Upon transfection, anhydrotetracycline (aTc) was continuously applied together with selection for the *bsd* marker to preserve conditional CLAG3 expression and avoid TetR-DOZI-aptamer mediated suppression. After parasite outgrowth under this selection, limiting dilution cloning yielded the *C3-TetR* clone. PCR and DNA sequencing confirmed faithful integration at the desired locus (S2 Fig).

We used immunoblotting to evaluate expression of the chimeric transgene product. With a CLAG3 antibody that recognizes the protein’s C-terminus in all examined *P*. *falciparum* lines, lysates from *C3-TetR* cultures yielded a band of somewhat slower mobility than observed in the KC5 parent, consistent with an expected increase of ~ 4 kDa based on addition of a C-terminal HA epitope tag and a larger variant motif on Dd2 CLAG3.1 (Fig 2B). When probed with anti-HA antibody, *C3-TetR* yielded a band of indistinguishable size but absent from the KC5 parent, as predicted by our transfection strategy (Fig 2C).

Immunoblotting with these antibodies also revealed robust CLAG3 knockdown upon *C3-TetR* cultivation without aTc (Fig 2B and 2C). Quantification of band densities from independent trials revealed an approximately 90% knockdown (Fig 2D). Immunofluorescence microscopy confirmed homogeneous knockdown, as expected for clonal parasites carrying the conditional regulation cassette (Fig 2E). Despite effective knockdown, we could propagate *C3-TetR* without aTc indefinitely and did not detect reduced growth under standard *in vitro* conditions.

We next examined the effect of transfection and CLAG3 knockdown on PSAC phenotypes and measured osmotic lysis kinetics in sorbitol, a sugar alcohol taken up by infected cells primarily via PSAC [45]. While 15 μM ISPA-28 had negligible effect on uptake into KC5-infected cells, channels in *C3-TetR* were potently blocked by ISPA-28, with affinity comparable to that seen in Dd2 and in other transfections using the Dd2 *clag3*.*1* gene [37, Fig 2F–2H], as expected. Remarkably, however, aTc removal to knockdown CLAG3.1 only modestly reduced ISPA-28 sensitivity despite a near-complete knockdown of the block-enabling transgene (Fig 2D and 2E). Although we could detect a modest reduction in ISPA-28 sensitivity (red vs. blue triangles, Fig 2G; *P* = 0.005 in comparisons at a 15 μM concentration, *n* = 7 independent trials each), the effect of knockdown was much less than expected by models invoking a single CLAG3 monomer that directly forms a pore. Retained ISPA-28 sensitivity despite a ~90% knockdown of the Dd2 CLAG3.1 protein is surprising because this inhibitor is inactive against channels associated with all other CLAG proteins in KC5 and its parental lines (Fig 2F and S1B Fig); this result indicates that a remarkably low dose of the Dd2 CLAG3.1 protein can confer ISPA-28 block on these parasite-induced channels.

A direct 1:1 relationship between CLAG3 and channel activity was also contradicted by measurements of PSAC-mediated permeability, which is inversely proportional to the inhibitor-free osmotic lysis halftime [46]. The halftime for KC5 parasites in sorbitol, 7.7 min, corresponds to an apparent sorbitol permeability of 0.13 ± 0.007 min^-1^, which is similar to values obtained with other wild-type parasites [45]; osmotic lysis of *C3-TetR*-infected cells was significantly slower both with and without aTc addition (Fig 2H). Although the reduced permeability without removal of the aTc stabilizer might reflect expression of an altered CLAG3 protein, we consider incomplete protection of translation by aTc to be a more conservative explanation. Experiments using parasites cultured with a higher aTc concentration of 4 μM did not significantly affect transport rates, but were limited by increasing toxicity of aTc on parasite cultures. Another possibility, *clag3* and *clag2* silencing due to transfection under blasticidin S selection [24,25,47], could also contribute to reduced *C3-TetR* permeabilities. Because *clag* gene silencing and transport phenotypes recover quantitatively within 4 weeks of blasticidin S removal [47], a significant effect of drug selection is unlikely. Surprisingly, cultivation of *C3-TetR* without aTc produced only a modest reduction in the lysis halftime that did not reach statistical significance (*P* = 0.7, *n* = 7 trials each), again inconsistent with a 1:1 relationship between CLAG3 expression and PSAC formation.

We also transfected 7G8 parasites with *pBAC-Dd2C3-TetR-DOZI*, obtained an integrant clone by limiting dilution, and observed similar changes in transport phenotypes that further argue against a simple stoichiometric relationship between CLAG3 expression and PSAC formation (S3 Fig). PCR indicated that the *7G8-TetR* limiting dilution clone had undergone homologous recombination of the plasmid into the 7G8 parasite’s *clag3*.*1* gene while preserving the native *clag3*.*2* gene (S3A and S3B Fig). Immunoblotting with anti-HA epitope tag antibodies confirmed transgene expression that could be effectively abolished upon aTc removal; in contrast to the *C3-TetR* line, however, the total amount of CLAG3 was largely unaffected by conditional knockdown in *7G8-TetR*, as revealed with anti-CLAG3 antibodies that bind to a conserved C-terminal epitope (S3C and S3D Fig). RT-PCR revealed that the chimeric *clag3*.*1* gene is preferentially expressed in the presence of aTc and confirmed epigenetic switching to increase *clag3*.*2* expression by >100-fold upon conditional knockdown of *clag3*.*1* (S3E Fig); this switching accounts for the sustained production of CLAG3 in *7G8-TetR*. As with the *C3-TetR* clone, this transfectant also had preserved block by ISPA-28 despite quantitative reductions in the expression of the Dd2 *clag3*.*1* element required for ISPA-28 block (S3F and S3G Fig). Although still less than predicted by simple models of block, aTc removal reduced ISPA-28 efficacy to a greater extent in the *7G8-TetR* parasite than in *C3-TetR* (S3H and S3I Fig.), presumably because of switching and expression of the unmodified *clag3*.*2* gene associated with ISPA-28 insensitive channels.

### A viable CLAG3-null parasite has reduced, but partly preserved PSAC activity

In light of the modest effects of CLAG3 knockdown, we next explored whether a viable *clag3-*null parasite could be produced and used CRISPR/Cas9 transfection with a guide RNA that targets exon 1 of *clag3h* in KC5 and *clag3*.*2* in other *P*. *falciparum* lines. Although transfection has previously been used to knockout *clag3*.2 [40], the resulting parasite retained an intact *clag3*.*1* gene. While those authors used drug selection to silence the remaining *clag3*.*1* gene, low-level CLAG3 expression is likely with such silencing and would yield transport phenotypes similar to those described above for *C3-TetR*.

**Fig 3:**
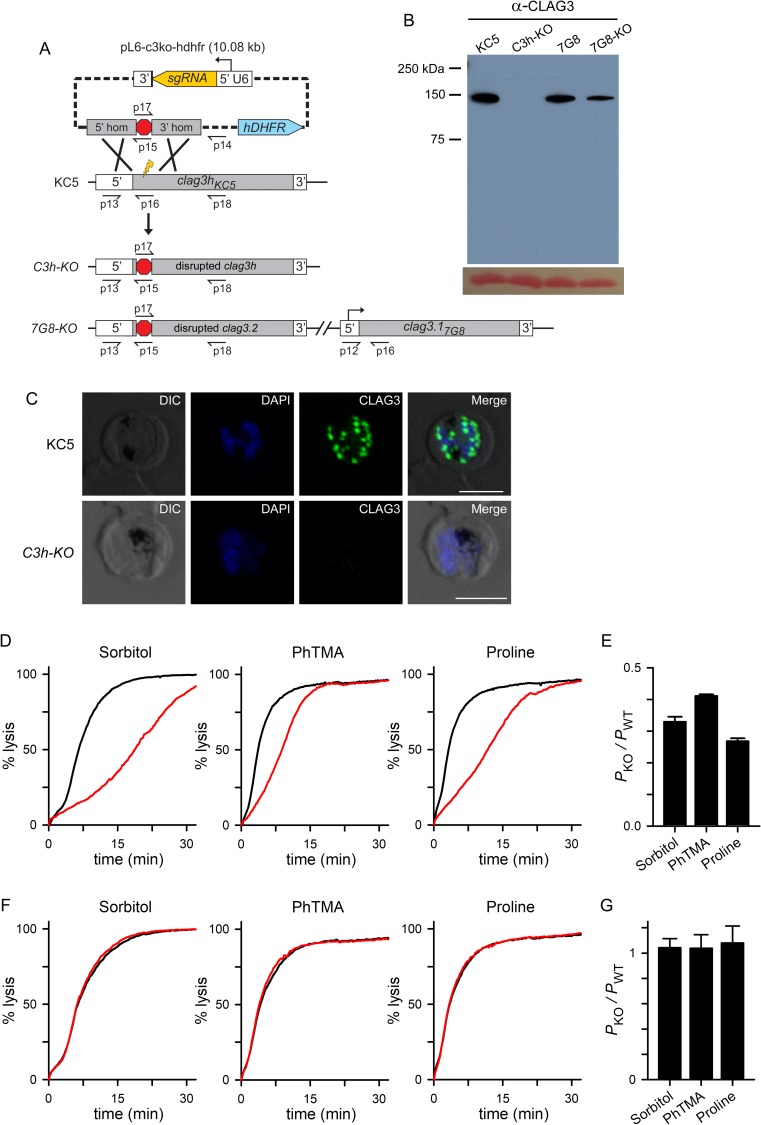
Production of a viable CLAG3-null parasite with incomplete reduction in solute permeabilities. (A) CRISPR/Cas9 transfection strategy for knockout of *clag3h* in KC5; the result of the transfection in 7G8, which carries two *clag3* paralogs, is also shown (bottom ribbon). Primer positions are shown as used for PCR results in S4 Fig; primer sequences are listed in S2 Table. (B) Anti-CLAG3 immunoblot using total cell lysates from indicated parasites; bottom shows Ponceau S hemoglobin staining as a loading control. (C) Indirect immunofluorescence images of schizont stage KC5 wildtype and *C3h-KO* parasites probed with anti-CLAG3 antibody (green). Scale bar, 5 μm. (D) PSAC-mediated osmotic lysis kinetics for indicated solutes. Black and red curves reflect kinetics for KC5 and *C3h-KO*. (E) Mean ± S.E.M. permeabilities of indicated solutes in the knockout line, normalized to 1.0 for matched experiments with the wild-type parental line (*P*_*KO*_/*P*_*WT*_). The residual permeabilities of these solutes differed from one another (*P* < 0.01, one-way ANOVA). (F) Preserved PSAC-mediated transport of each solute in *7G8-KO* when compared to 7G8 (red and black traces, respectively). (G) Mean ± S.E.M. permeabilities in *7G8-KO* relative to matched experiments with 7G8 confirming no detectable reduction in each solute’s transport (*n* = 3 trials each).

The KC5 line and a two-plasmid CRISPR/Cas9 strategy was used to address this possibility and avoid a dependence on gene silencing for the null phenotype (Fig 3A). We successfully generated the *C3h-KO* limiting dilution clone that lacks an intact *clag3* gene (S4A Fig). Immunoblotting and immunofluorescence imaging confirmed complete absence of CLAG3 protein in this parasite (Fig 3B and 3C).

We then examined PSAC-mediated transport and found significant, but incomplete reductions in the permeabilities of sorbitol, the organic cation phenyl-trimethylammmonium (PhTMA^+^), and proline, solutes representing the broad selectivity of PSAC (Fig 3D). Each solute’s permeability was reduced by 60–75%, as determined from the inverse relationship between permeability and time to osmotic lysis [46]; interestingly, ANOVA comparisons and post-hoc testing revealed differing relative decreases for each solute (Fig 3E, *P* < 0.01, *n* = 3–4 trials for each solute).

Transfection of the 7G8 parasite with the same *pL6-c3ko-hdhfr* plasmid yielded the *7G8-KO* line with a disrupted *clag3*.*2* gene (Fig 3A and S4B Fig). This knockout line retains a full length *clag3*.*1* gene that produces an unmodified CLAG3 protein (Fig 3B). Continued expression of this paralog resulted in unchanged permeabilities for sorbitol, PhTMA^+^, and proline (Fig 3F and 3G). Thus, *7G8-KO* serves an important control to show that loss of CLAG3 protein is responsible for the reduced permeability in *C3h-KO*. We have not pursued simultaneous disruption of both *clag3* genes in the 7G8 background; such a double knockout would have reduced PSAC activity quantitatively similar to that of *C3h-KO* because these lines have nearly identical sequences for RhopH2, RhopH3, and other CLAG proteins.

**Fig 4:**
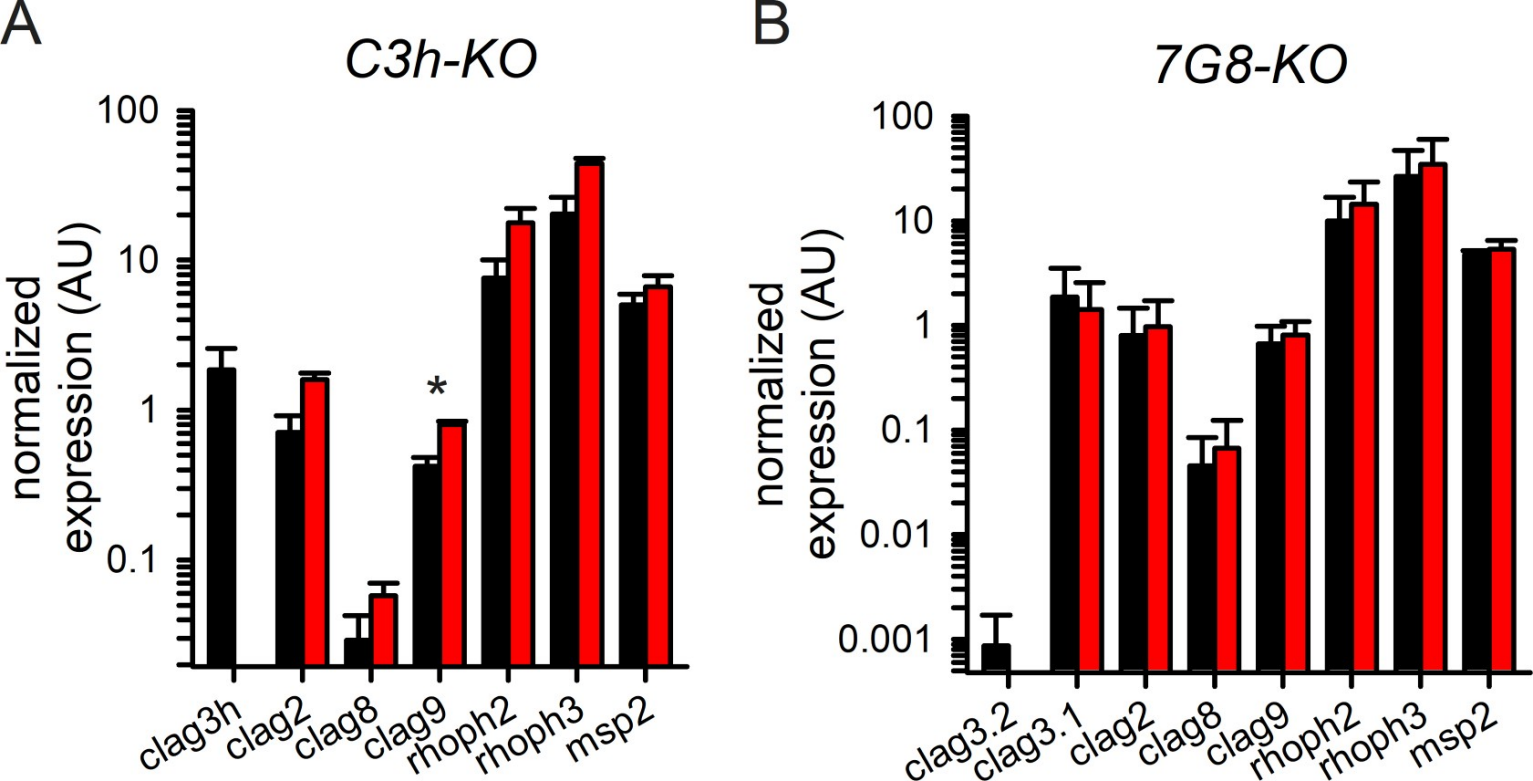
Modest effects on expression of *clag* paralogs and *rhoph* genes in the knockout lines. (A) Mean ± S.E.M. normalized expression of *clag* and *rhoph* genes in KC5 and *C3h-KO* (black and red bars, respectively; *n* = 3), calculated according to 2^-ΔCt^ using PF07_0073 as an internal control. Only *clag9* exhibited a statistically significant upregulation in the *Ch3-KO* knockout (asterisk, *P* = 0.007). (B) Insignificant changes in expression of these genes in the *7G8-KO* knockout when compared to 7G8 (red and black bars, respectively; *n* = 2–3 for each gene). The *msp2* control exhibits stage-specific transcription similar to *clag* and *rhoph* genes, and is not affected in these knockout lines.

While *clag* genes on other *P*. *falciparum* chromosomes have not been unambiguously implicated in PSAC activity [16], it is possible that successful production of a *clag3* null parasite and partly preserved host cell permeability result from compensatory increases in expression of these paralogs. We therefore used quantitative RT-PCR to measure transcript levels for *clag* paralogs, *rhoph2* and *rhoph3* in the knockout lines. In the KC5 wild-type parent, *clag3h* was found to be the most highly expressed member of the *clag* gene family; its knockout produced only modest changes in expression of other *clags*, *rhoph2*, and *rhoph3* (Fig 4A). Correcting for family-wise errors that result from multiple comparisons, the increase in *clag9* expression, but not those of *rhoph3* and *clag2*, reached statistical significance (*P* = 0.007, 0.026 and 0.028, respectively, Student’s *t* test with the Holm-Bonferroni correction; *n* = 3 independent trials each); *clag8* and *rhoph2* were not significantly upregulated in *C3h-KO*. None of these genes were upregulated in *7G8-KO* (Fig 4B). Thus, both lines tolerate *clag3* knockout without marked compensatory changes in other genes linked to PSAC.

Preferential expression of *clag3*.*1* in the 7G8 line results from unbalanced switching rates between the two *clag3* paralogs, as previously reported [12,30]. Because the *7G8-TetR* conditional knockdown confirms competence for epigenetic switching (S3E Fig), we did not pursue production of a *clag3*.*1* knockout in the 7G8 background: that transfectant would also retain unaltered transport properties without changes in other *clag* and *rhoph* genes.

### *C3h-KO* requires supraphysiological levels of essential nutrients

**Fig 5:**
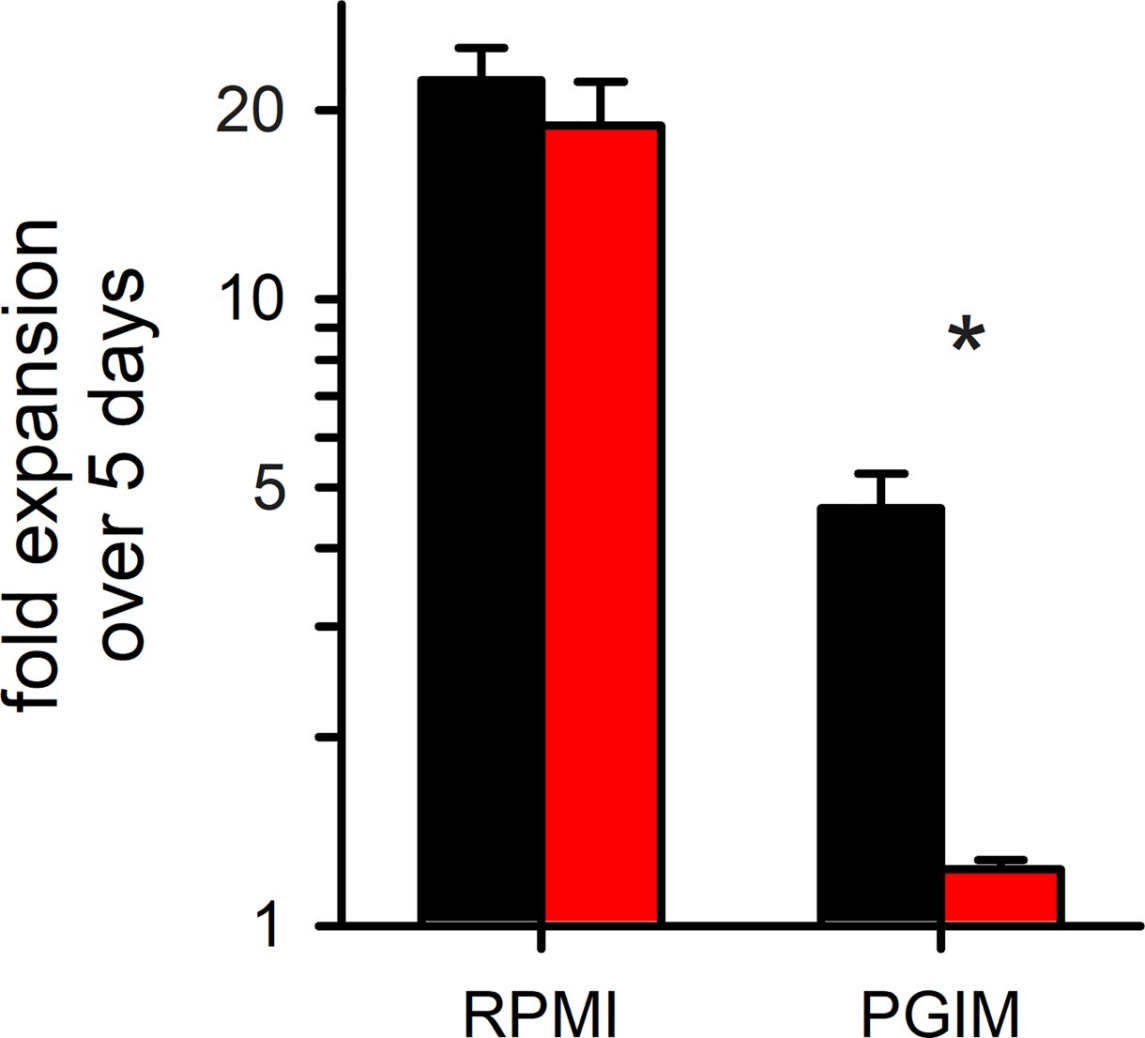
Comparison of *C3h-KO* and wild-type parasite growth rates. Mean ± S.E.M. expansion of wild-type and *C3h-KO* cultures over 5 days (black and red bars, respectively; *n* = 3). Expansion in indicated media is shown on a logarithmic scale. Asterisk, *P* = 0.006.

Although transfection to produce *C3h-KO* indicates that CLAG3 is not needed for *in vitro* propagation, the conservation and variable expansion of the *clag3* clade in *Plasmodium spp*. suggest a more essential role under *in vivo* conditions in vertebrate infections [27,29,33]. In light of PSAC’s role in nutrient uptake [14], we hypothesized that the high concentrations of most nutrients in standard RPMI 1640-based media may permit growth of the CLAG3-null parasite but that the lower levels in host plasma may prove inadequate. We therefore compared growth in RPMI 1640-based media to that in PGIM, a modified medium with more physiological concentrations of key nutrients [14]. Using SYBR Green to measure nucleic acid production, 5 day expansion of *C3h-KO* was indistinguishable from that of its wild-type parent when cultures were maintained in RPMI 1640-based media (Fig 5; *P* = 0.47, *n* = 3 trials with triplicate measurements). In PGIM, however, *C3h-KO* growth was drastically compromised, with nucleic acid production reduced by 17 ± 7 fold when compared to that of the KC5 parent in the same medium (*P* = 0.006, *n* = 3). While wild-type cultures also grow slower in PGIM than in RPMI 1640 (compare black bars, Fig 5), their PGIM expansion rates match those seen with pooled human serum [14], suggesting that this modified medium better reflects *in vivo* parasite expansion than RPMI 1640-based media. Although circulating nutrient levels in human subjects are difficult to measure and standardize between laboratories [48,49], the nominal concentrations of isoleucine and hypoxanthine in PGIM more closely resemble levels in healthy human volunteers. Plasma levels for these and other nutrients have not been accurately measured in malaria-endemic countries, but limited access to quality nutrition suggests that they may be lower. Thus, while we successfully produced a viable CLAG3-null parasite under *in vitro* culture conditions, our findings suggest that CLAG3 is required for *P*. *falciparum* survival and expansion in human infections, where parasite nutrient acquisition is rate-limiting.

### Inhibitors also support direct CLAG3 contribution to PSAC formation

**Fig 6:**
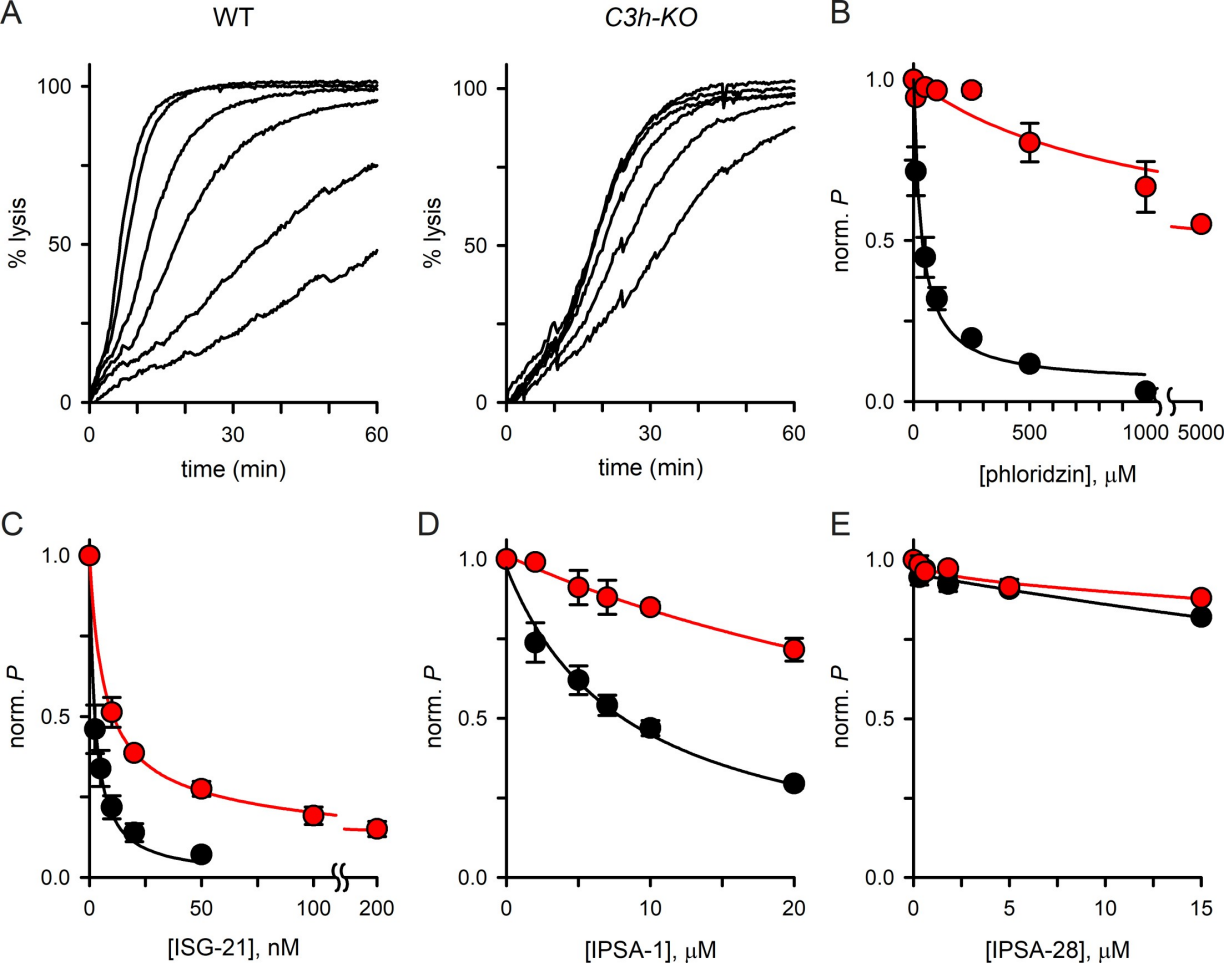
Altered pharmacology in *C3h-KO*. (A) Sorbitol-induced osmotic lysis kinetics for wild-type and *C3h-KO*. Traces are shown for cells suspended with the inhibitor phloridzin (0, 10, 50, 100, 250, and 500 μM in the wild-type panel; 0, 100, 250, 500, 5000 μM for *C3h-KO*). Increasing inhibitor concentrations produce monotonic increases in transport block (top to bottom traces in each panel); note that there is less effective block of *C3h-KO* channels despite use of higher [phloridzin]. (B—E) Mean ± S.E.M. residual permeabilities from dose response experiments as in A for phloridzin, ISG-21, ISPA-1, and ISPA-28, respectively. In each panel, wild-type parent and *C3h-KO* are shown with black and red circles, respectively (up to 4 trails at each concentration). Solid lines represent best fits to *y = a*/(1 + (*x*/*b*)) + (1-*a*)/(1 + (*x*/*c*)).

Finally, we examined PSAC block by known small molecule inhibitors and found that the *C3h-KO* knockout line exhibits marked changes in pharmacology. Phloridzin, a nonspecific, low affinity inhibitor that acts at the intracellular face of the channel [50,51], was markedly less effective in inhibiting sorbitol uptake in *C3h-KO* than in the wild-type parent (Fig 6A and 6B; half-maximal blocking concentrations, *K*_*0*.*5*_, of 4200 ± 1100 and 45 ± 10 μM, *P =* 0.01, *n* = 4 dose responses each). ISG-21, a potent and specific inhibitor found through high-throughput screening that appears to act at an extracellular site on the channel [14], also exhibited a significantly reduced efficacy in the CLAG3 knockout (Fig 6C, *K*_*0*.*5*_ of 15.0 ± 2.4 nM vs. 2.6 ± 0.7 nM in the wild-type parent, *P* = 0.008, *n* = 3). ISPA-1, the inhibitor used to map the *clag3* locus in the 7G8 x GB4 cross (Fig 1), was also less effective (Fig 6D, *P* < 0.001, *n* = 3), further supporting direct interaction with CLAG3. Finally, consistent with its specificity for the Dd2 CLAG3.1 protein, knockout of CLAG3 did not alter the lack of ISPA-28 activity against channels on the KC5 line (Fig 6E); this finding also further excludes ISPA-28 blocking activity against channels linked to *clag* paralogs on other chromosomes. These complex changes in pharmacology in the *C3h-KO* knockout parasite support a critical role of CLAG3 in establishing and modifying PSAC activity at the host membrane.

## Discussion

Here, we used linkage analysis and distinct reverse genetic approaches to study the increased permeability of erythrocytes infected with malaria parasites. Described initially with macroscopic flux measurements [52,53], these changes are mediated by the broad-selectivity plasmodial surface anion channel (PSAC) and are conserved on human and other vertebrate erythrocytes infected with malaria parasites [19,54]. PSAC activity is essential for intracellular parasite growth and replication as it functions in nutrient uptake from host plasma [14,20]. Although originally proposed to result from upregulation of transporters endogenous to the erythrocyte, many studies have now linked PSAC activity to a parasite-encoded RhopH protein complex consisting of CLAG3, RhopH2, and RhopH3 [12,20,21,37]. The present studies provide three new insights into the role of CLAG3 in PSAC formation and intracellular growth of malaria parasites. First, we used DNA transfection to produce the first CLAG3-null parasite and show that loss of CLAG3 does not compromise growth under standard *in vitro* culture conditions; growth in PGIM, a modified medium that more closely resembles *in vivo* nutrient availability [14], was dramatically compromised, implicating CLAG3 requirement for parasite survival and expansion in human infections. Second, we found that loss of CLAG3 compromises but does not abolish PSAC activity, which is surprising given the multiple lines of evidence supporting this protein’s central role and our genetic mapping studies (Fig 1). Third, we used simultaneous CLAG3 allele replacement and conditional knockdown to determine that remarkably low levels of CLAG3 protein can confer block by ISPA-28, a PSAC inhibitor specific for the engineered transgene. In addition to providing independent evidence for a CLAG3 role in channel-mediated nutrient uptake, our studies reveal the complex behavior of knockdown and knockout lines despite simple Mendelian inheritance in a *P*. *falciparum* cross. We propose a conservative model that accounts for these and other observations from previous studies.

**Fig 7:**
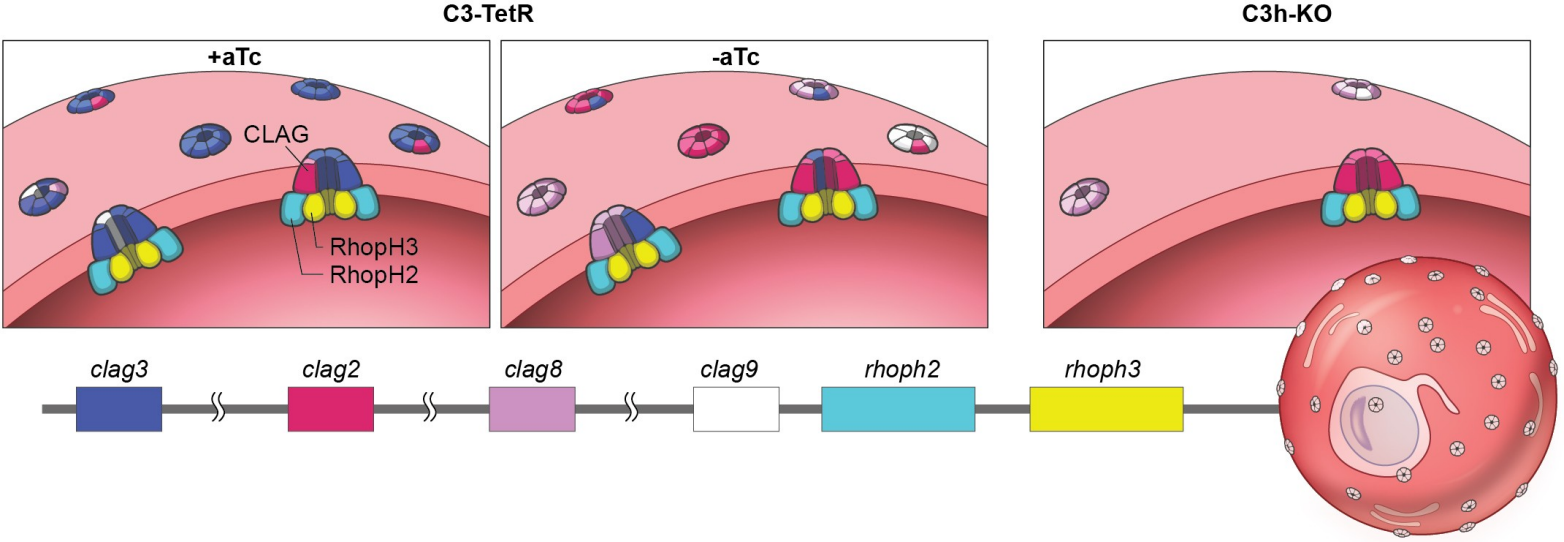
Proposed model for CLAG3 contribution to increased permeability of infected erythrocytes. Illustration shows the host erythrocyte membrane with channels composed of complexes that have several CLAG monomers in association with RhopH2 and RhopH3 proteins at their cytosolic face. Our model incorporates the observed 90% reduction of CLAG3 protein in *C3-TetR* when aTc is removed (middle vs. left panel), the 60–75% reduction in permeability in *C3h-KO* (right panel), and the greater transcription of *clag3* genes than of other *clags*. We propose that ISPA-28 block of channels in the *C3-TetR* conditional knockdown is preserved because channel oligomers that contain at least one CLAG3 subunit retain sensitivity to this inhibitor; the precise stoichiometries of CLAG, RhopH2, and RhopH3 subunits and their relative positions within functional channels remain unknown. The *C3h-KO* knockout retains PSAC activity at a reduced level because other CLAG paralogs may oligomerize to form channels. Ribbon legend shows subunit color-coding with breaks to reflect expression from distinct chromosomes.

While loss of RhopH2 or RhopH3 abolishes PSAC activity in sensitive whole-cell patch-clamp measurements [20], our CLAG3-null parasite retains 25–30% of the wild-type permeability. Does this suggest that RhopH2 and RhopH3 are more important PSAC subunits? What mechanisms might account for the differing effects of individual subunit knockdowns? In one model, CLAG3 plays a lesser role in PSAC formation while RhopH2 and RhopH3 serve strictly essential roles, possibly contributing directly to the nutrient pore. This model is exemplified by maxi-K potassium channels, where the pore is formed entirely by α subunit oligomers but a non-essential β subunit remains associated with the channel to serve important roles in activation at the cytoplasmic face [55]. In contrast to this model’s predictions for PSAC, protease susceptibility experiments have revealed that CLAG3 spans the host membrane while RhopH2 and RhopH3 are primarily or exclusively intracellular [15,20]. Furthermore, computational analyses, various inhibitor binding studies and site-directed mutagenesis all suggest CLAG3 carries a critical pore-lining transmembrane domain and argue against a non-essential accessory role for CLAG proteins [15,23]. To incorporate these findings, we propose an alternate model wherein CLAG proteins primarily form the channel pore while RhopH2 and RhopH3 serve essential, but poorly understood functions at the channel’s intracellular face (Fig 7). This model posits that CLAG3-null parasites still make functional channels because other CLAG paralogs derived from *clag2*, *clag8*, or *clag9* gene expression can associate with RhopH2 and RhopH3 to allow pore formation [16,56]. Because *rhoph2* and *rhoph3* are single copy genes in all *Plasmodium spp*., their knockouts cannot be compensated in a similar fashion and are, therefore, unable to sustain nutrient uptake.

Further supporting a critical role for CLAG proteins is the observation that *clags* appear to be the only multigene family strictly conserved throughout *Plasmodium spp*. [16,57–59]. Family size varies significantly with some plasmodial species having only two *clags* and others having up to 35 copies [29]. Recent studies in the human *P*. *falciparum* pathogen suggest ongoing expansion of this family [27], with active evolution of diversifying mutations in the two *clag3* genes [24]. It is not clear why this family has undergone variable expansion and why only some members are under epigenetic control [30]. One possibility is evasion of host immunity targeting the surface-exposed domain on CLAG proteins [12]. Several observations are consistent with this explanation. RhopH2 and RhopH3, PSAC components that are not surface-exposed (Fig 7), can serve their essential functions as single-copy genes without expansion. Immune evasion is also supported by the highly variant sequence at the exposed CLAG3 domain, which appears to be under immune selection [34,60]. Indeed, ISPA-28 and ISPA-1 were identified as inhibitors specific for certain CLAG3 alleles because these compounds block PSAC through interactions with this variant region. An important problem with the immune evasion proposal, however, is that 2 or 3 paralogs, as present in many *Plasmodium spp*., are generally considered insufficient to allow repeated cycles of switching and immune evasion [32].

An interesting alternative hypothesis is that *clag* gene expansion allows for production of distinct channels with solute selectivities that fine-tune nutrient uptake; this fine-tuning would be advantageous as nutritional status may vary significantly amongst humans and other hosts. Distinct channel subpopulations may also facilitate uptake of diverse nutritive solutes while still maintaining a low Na^+^ permeability and erythrocyte osmotic stability [61,62]. Supporting this hypothesis, a recent study found preferential expression of *clag3*.*2* in parasites drawn from humans with malaria; some, but not all, of these parasites switched to *clag3*.*1* upon *in vitro* culture, suggesting regulation by extracellular nutrient availability [33]. This possibility is also supported by their observation that low concentrations of blasticidin S, a toxin that enters infected cells through PSAC [47], selects for *clag3* switching in some, but again not all, lines [25,33]. Counter to this hypothesis, our transport measurements have not revealed significant changes in solute selectivity upon *clag3* expression switching (data for *7G8-TetR* in S3 Fig and data in [12]). In the present studies, *clag3* knockout reduced permeability of sorbitol, proline and the organic cation PhTMA^+^ by similar amounts (Fig 3D and 3E), further countering the proposal that *clag3*-associated channels transport specific solutes while other solutes are acquired by channels linked to *clag2*, *clag8* or *clag9*.

Our CLAG3-null parasite grows without detriment in a standard RPMI 1640 medium that contains supraphysiological concentrations of amino acids and vitamins [48]. Remarkably, however, *C3h-KO* growth was largely abolished in PGIM, a modified medium with more physiological levels of key nutrients (Fig 5). As parasite growth rates in PGIM match those in pooled human serum without addition of synthetic media products [14], our findings suggest that CLAG3 is needed under *in vivo* conditions but dispensable under nutrient-rich culture conditions. PGIM may also underestimate the *in vivo* efficacy of inhibitors that target parasite nutrient acquisition because it has unchanged, high levels of many nutrients acquired via PSAC [48]. Because *in vitro* parasite growth is compromised for many of these permeant nutrients in single solute elimination studies [63], we suspect that various other modifications of the RPMI 1640 formulation to reduce key nutrients to more physiological levels would also compromise or abolish growth of the CLAG3-null parasite. As no modified medium can confidently replicate the conditions encountered in human infections, we propose that ongoing drug discovery targeting PSAC should be guided by *in vivo* studies of transport rates and parasite survival and propagation. As potent inhibitors have only been validated under *in vitro* conditions to date [14,64], these inhibitors may prove even more efficacious in future animal and human infection studies.

While conditional knockdown has been primarily used to study genes refractory to knockout [41,65], our combined use of knockdown and knockout strategies provided important insights not possible with either approach alone. Our knockdown studies reveal that remarkably low doses of CLAG3, such as those that persist in previously generated silencing mutants and conditional knockdowns [24,40], are sufficient to preserve PSAC phenotypes. Combined use of conditional knockdown and allele replacement to confer ISPA-28 sensitivity permitted sensitive detection of this dose effect on channel phenotypes in our study. The observed retention of ISPA-28 sensitivity despite a 90% knockdown of transfected CLAG3 allele is inconsistent with models invoking a single CLAG3 subunit per functional channel because this arrangement would yield a direct linear relationship between transgene expression and ISPA-28 potency. Higher order stoichiometries, e.g. two or more CLAG molecules per channel, can produce retained ISPA-28 sensitivity despite quantitative knockdown if we assume that CLAG2, CLAG8 and/or CLAG9 also produce channels and that CLAG paralogs aggregate randomly to form functional channels in the host membrane. In general, if *k* CLAG molecules are required to form the channel, the number of distinct channels that can be formed from combinations of the four CLAG paralogs in the *C3-TetR* parasite is given by the binomial coefficient $\left(\begin{array}{c} 3+k\\ k \end{array}\right)$; this corresponds to 10, 20, and 35 distinct complexes that could be formed with 2, 3, or 4 CLAG molecules per channel, respectively. As the number of possible complexes increases, the fraction that contain one or more CLAG3 monomers also increases: 40, 50, and 57.1% of channels will contain at least one CLAG3 monomer in dimeric, trimeric, and tetrameric arrangements, respectively, if each of the paralogs is equally abundant. (If CLAG3 is present at higher copy number than other paralogs, as our data suggests, then this fraction will increase even more rapidly with subunit stoichiometry.) Now, if channels containing one or more CLAG3 monomers are blocked by ISPA-28 with similar affinities, then ISPA-28 block will be preserved to greater extents in the *C3-TetR* CLAG3 knockdown for higher stoichiometries (Fig 7). Thus, our pharmacological studies using the *C3-TetR* knockdown line exclude models where a single CLAG monomer directly contributes to PSAC formation; instead, they implicate higher order complexes at the host membrane and provide experimental evidence for contributions from CLAG paralogs encoded from other *P*. *falciparum* chromosomes. Larger complexes are also supported by the recognition that a 1:1:1 complex of CLAG3, RhopH2, and RhopH3, as suggested by immunoprecipitation studies [66], lacks the number of predicted transmembrane domains generally needed to form a stable aqueous pore in biological membranes [16,26]. Anomalous migration of these membrane proteins in native PAGE experiments, as often seen for integral proteins [67], has prevented an accurate size determination for this complex [37]. While coimmunoprecipitation has detected associations between two CLAG3 isoforms in a transfected parasite [37], our transport measurements provide the first experimental evidence for functional oligomerization to form the nutrient channel at the host membrane.

Our observations on knockdown phenotypes parallel those in another pharmacological study that expressed mixtures of sensitive and resistant alleles of K^+^ channel subunits [68]; by varying the amounts of the two alleles in a heterologous expression system, that study correctly determined that only a tetrameric arrangement of the K^+^ channel could account for their observed phenotypes. Unfortunately, several complexities intrinsic to our work prevented us from a similar precise determination of the number of CLAG subunits required to form a channel. When compared to this K^+^ channel study, the larger number of CLAG paralogs and an inability to control their amounts with the precision of a heterologous expression system are obvious problems for precise quantification. Additionally complicating is that the level of ISPA-28 block depends on the precise amounts of each CLAG paralog faithfully exported and delivered to the host membrane; we are unable to estimate this with any accuracy but recognize that it is only partly determined by each gene’s relative transcription (Fig 4). Another unknown is that RhopH2 and RhopH3 also appear to contribute directly to the channel as they remain associated with CLAGs from the time of their co-translational assembly and rhoptry packaging up through their eventual insertion into the erythrocyte membrane [20]. Indeed, the stoichiometries and contributions of these other conserved proteins to PSAC phenotypes remain elusive.

Because enzymes that modify other proteins or lipids may also have largely preserved phenotypes upon quantitative knockdown, we also wondered whether our knockdown phenotype could reflect a model where CLAG3 does not directly contribute to the pore, but instead enzymatically activates other proteins to form the channel, as has been frequently proposed [69]. While such enzymatic or regulatory roles for CLAG3 remain formally possible, a point mutation introduced in the surface-exposed variant loop of the Dd2 CLAG3.1 allele dramatically alters PSAC’s protease susceptibility and ISPA-28 block [15]. Recognizing that ISPA-28 and other inhibitors block these channels instantly when added in whole-cell patch-clamp experiments performed with non-physiological solutions [12,51], we consider an enzymatic or regulatory CLAG3 role unlikely: we are unaware of any precedent for such a model in the electrophysiology literature.

The model proposed here conservatively accounts for the phenotypes of our CLAG3-null and conditional knockdown lines as well as those from prior studies. Nevertheless, refinements or new structure-function models remain an important direction for future molecular and biochemical studies, which are critical for understanding host cell remodeling and for advancing antimalarial development against parasite nutrient acquisition.

## Materials and methods

### Parasite culture

*P*. *falciparum* laboratory lines were cultivated at 37 ^o^C under 5% O_2_, 5% CO_2_, 90% N_2_ in O^+^ human erythrocytes obtained from Interstate Blood Bank (Memphis, TN) at 5% hematocrit in RPMI 1640 medium (KD Medical) supplemented with 25 mM HEPES, 50 μg/mL hypoxanthine, 0.5% NZ Microbiological BSA (MP Biomedicals), gentamicin and 28.6 mM NaHCO_3_ (Gibco). A modified medium, PGIM [14], follows the RPMI 1640 medium with supplements but has reduced concentrations of isoleucine and hypoxanthine (11.4 μM and 3.01 μM, respectively). Primers specific to 5’ and 3’ UTR regions of *clag3*.*1* and *clag3*.*2* were used to detect the number and possible combinations of *clag3* alleles in the GB4 and 7G8 parental lines (S2 Table; SpeedSTAR HS DNA polymerase, Takara).

### Osmotic lysis measurements

The increased permeability of infected erythrocytes to organic solutes and block by strain-specific inhibitors was quantified using a kinetic assay that tracks 700 nm light transmittance through a suspension of cells [45]. Osmotic lysis resulting from uptake of sorbitol, a sugar alcohol with high PSAC permeability, produces increased light transmittance. Synchronized parasite cultures were enriched at the trophozoite stage with the Percoll-sorbitol method, washed, and resuspended at 37 ^o^C in lysis buffer (280 mM sorbitol, 20 mM Na-HEPES, 0.1 mg/ml bovine serum albumin, pH 7.4) to initiate sorbitol uptake and cell swelling that leads to osmotic lysis. Iso-osmotic replacement of sorbitol with either 280 mM proline or 145 mM PhTMA^+^Cl^-^ was used to measure permeabilities of these solutes; these solutes are representative of the broad range of solutes with PSAC permeability [62]. Inhibitors, where present, were added from DMSO stock solutions without preincubation; control experiments excluded DMSO effects on uptake measurements. 700 nm light transmittance was then continuously tracked in a DU640 or DU800 spectrophotometer (Beckman Coulter). Inhibitor dose-responses and reductions in permeability in transfectant parasites were then calculated from the time required to reach a fractional lysis threshold, based on a conservative two-compartment model of infected cell osmotic lysis in permeant solutes [46]. This method produces estimates of permeability coefficients and inhibitor affinities that quantitatively match those obtained with patch-clamp methods [70].

ISPA-1 was identified as a strain-specific PSAC inhibitor in prior high-throughput screens using four clonal laboratory strains of *P*. *falciparum* (Dd2, HB3, 3D7, and Indo 1; [12]). Transport surveys identified this compound as suitable for genetic mapping in the GB4 x 7G8 cross. Dose response studies revealed that ISPA-1 block was adequately fitted by a single Langmuir isotherm with normalized sorbitol permeability, *P*, given by *P* = *a*/ [1 + (*x*/*b*)] where *a* and *b* are constants. A 10 μM concentration of ISPA-1 produced the greatest difference in parental phenotypes and was used to examine block in progeny clones. Dose responses for block by ISPA-28, a strain-specific inhibitor with potent and specific block of channels linked to the Dd2 *clag3*.*1* gene [12], required fitting to a sum of two Langmuir isotherms, as reported previously [37].

### QTL analysis

Associations between ISPA-1 block of solute uptake and 290 known microsatellite markers from the GB4 x 7G8 cross were sought by quantitative trait loci (QTL) analysis [36]. QTL analysis was performed using the multiple imputation algorithm and R/qtl software (freely available at http://www.rqtl.org/) with conditions suitable for haploid asexual parasite crosses, as described [71]. A significance threshold of *P* = 0.05 was calculated from 1000 permutations. Secondary scans after removing the effects of the *clag3* locus did not identify additional contributing genomic loci. DNA sequencing and examination of the publicly available SNP database for *P*. *falciparum* genetic crosses (https://www.malariagen.net/apps/pf-crosses/1.0/#variants) did not reveal mutations in the *clag3* genes of progeny clones relative to their corresponding parental lines.

### Cloning and transfections

The *C3-TetR* and *7G8-TetR* conditional knockdown clones were produced through allelic exchange by single homologous recombination with *pBAC-Dd2C3-TetR-DOZI*, which was prepared by subcloning of a 3.2 kb fragment of the Dd2 *clag3*.*1* gene from the pHD22Y-120w-flag-PG1 vector [12]. This bacmid was propagated in BAC-Optimized Replicator v2.0 electrocompetent cells (Lucigen) in TB medium with 50 μg/mL kanamycin and 1x arabinose induction, per the manufacturer’s recommendations. Transfected cultures were selected with 2.5 μg/mL blasticidin S and maintained on 2 μM anhydrotetracycline (aTc) to preserve expression of the targeted gene product [42].

CLAG3 knockout lines were produced by CRISPR/Cas9 transfection using the *pL6-c3ko-hdhfr* vector. In-Fusion cloning (Clontech) was used to introduce the sgRNA for expression under the *Pf*U6 promoter. A synthetic DNA construct carrying homology arms, shield mutations at the Cas9 target site and in-frame stop codons that disrupt translation was also inserted by In-Fusion cloning. Cas9 was expressed from an unmodified pUF1-Cas9 plasmid [72]. After transfection, cultures were selected with 1.5 nM WR99210 (Jacobus) and 1.5 μM DSM-1 (BEI Resources) to ensure retention of plasmids.

All plasmids were confirmed with DNA sequencing and restriction digestion prior to transfection, which was initiated by loading uninfected erythrocytes through electroporation and subsequent parasite cultivation. After parasite growth was detected with Giemsa-stained slides, PCR was used to evaluate integration; limiting dilution clones were obtained by the c-SNARF method [73].

### Immunoblots

Trophozoite-stage cultures were enriched by the Percoll-sorbitol method to yield 96–99% infected cells and permit equivalent loading of synchronous parasites. Cells were lysed in chilled hypotonic lysis buffer (7.5 mM NaHPO_4_, 1 mM EDTA, pH = 7.5) with 2 mM PMSF prior to separation by SDS-PAGE (4 to 15% Mini-Protean TGX gel, Bio-Rad) and transfer to nitrocellulose membrane for immunoblotting. After blocking, primary antibodies were applied overnight in blocking buffer (anti-CLAG3, 1:2000 dilution; anti-HA tag, 1:1000 dilution, EMD Millipore). After washing to remove unbound antibody, horseradish peroxidate (HRP)-conjugated secondary antibody was applied (anti-mouse IgG, 1:10,000 dilution, Sigma-Aldrich) with Clarity Western ECL substrate (Bio-Rad). Binding was detected on Hyblot X-ray film. Band intensities were estimated from three independent trials using ImageJ software (https://imagej.nih.gov/) and normalization for hemoglobin loading with Ponceau S staining.

### Immunofluorescence Assays

Indirect immunofluorescence confocal microscopy was performed using thin smears prepared from parasite cultures. Dried slides were fixed in a chilled 1:1 acetone:methanol mixture for 5 min prior to blocking with 3% skim milk in PBS for 1 h at RT. Slides were incubated with mouse anti-CLAG3 (1:100 dilution) or mouse anti-HA (1:500, Sigma Aldrich) for 1 h at RT, washed with ice-cold PBS, and post-incubated with 2 μg/uL DAPI (4’,6-diamidino-2phenylindole) and goat anti-mouse AF488 at 1:500 dilution for 30 mins at RT. After washing in ice-cold PBS for 5 min, slides were then mounted with Prolong Diamond anti-fade mountant (Molecular Probes). Images were collected using a 64x oil immersion objective on a Leica SP5 or SP8 confocal microscope and processed using Leica LAS X software.

### Quantitative Real Time PCR

Expression of *rhoph* genes in knockout lines was quantified with real-time PCR using total RNA harvested 27 h after sorbitol synchronization of cultures and the PureLink RNA minikit (Ambion). Microscopic examination of smears confirmed harvest of matched, late trophozoite-stage infected cells.

Genomic DNA was removed by DNase I treatment (TURBO DNA-free kit, Ambion) prior to first-stand cDNA synthesis by reverse transcription using Superscript III (Invitrogen), oligo(dT) primers, and ~ 1.5 μg RNA from each parasite. The resulting cDNA was diluted and used for qRT-PCR with the QuantiTect SYBR Green kit (Qiagen). Primers were designed based on specificity for individual genes and a desired amplicon size of ~120 bp (S2 Table). qRT-PCR was carried out using the iCycler IQ multicolor real-time PCR system (Bio-Rad) and a three-step protocol: denaturation at 95°C for 15 min followed by 40 cycles of annealing at 52°C and extension at 62°C for 30 s each. The final stage used gradual heating from 55 to 95°C with 0.5°C steps over 30 s; this dissociation protocol was used to confirm the specificity of primer binding and product synthesis. Each qRT-PCR was accompanied by a negative control without reverse transcriptase to exclude gDNA contamination. All reactions were performed in triplicate with Pf07_0073 used as a constitutively expressed loading control; *msp2* was included as a transcription control as it exhibits stage-specific expression similar to *clag* and *rhoph* genes. The average threshold cycle (C_T_) values from each experiment were used to calculate expression according to 2^(mean C_T_ value of Pf07_0073 –mean C_T_ value of the gene of interest). Expression is presented in arbitrary units as the mean ± S.E.M. of results from independent RNA harvests.

### Parasite growth rates

Parasite expansion rates in RPMI 1640 and PGIM media were measured using a SYBR Green I-based fluorescence assay, as described previously [14]. Synchronous ring-stage cultures were seeded in 96-well microplates at 0.2% parasitemia and 2.5% hematocrit. Media was replaced after 48 h and cultures gassed daily. After cultivation for a total of 5 days, cells were lysed in buffer (20 mM Tris pH 7.5, 10 mM EDTA, 1.6% Triton X-100, 0.016% saponin, pH 7.5) with SYBR Green I nucleic acid gel stain (ThermoFisher) at 2500-fold dilution. After a 30 min RT incubation in the dark, fluorescence measurements were used to quantify parasite DNA (excitation, 485 nm; emission, 528 nm; BioTek Synergy HT reader). Expansion was estimated from triplicate wells after normalization to matched cultures killed by treatment with 20 μM chloroquine.

### Statistical analysis

All numerical data were calculated and plotted as mean ± S.E.M. from at least three trials. Statistical significance was calculated by unpaired Student’s *t-*test or one-way ANOVA tests as indicated. The Holm-Bonferroni method was used to correct for the family-wise error rate arising from multiple comparisons [74].

## Supporting information

S1 Fig CLAG3 sequence alignment and ISPA-28 binding site. (A) Multiple sequence alignment of indicated CLAG3 sequences from Dd2, 7G8, and GB4 lines. The C-terminal fragment expressed by the last exon of *clag3* is shown; CLAG3 sequences upstream of this region are also highly conserved. A single hypervariable region (HVR) in CLAG3 proteins and the region used for production of an anti-CLAG3 polyclonal antibody are both labeled. (B) Sorbitol-induced osmotic lysis kinetics for 7G8 and GB4 lines with 0, 0.3, 0.6, 1.8, 5, or 15μM ISPA-28 (top to bottom, respectively in each panel). Right panel shows mean ± S.E.M. permeability remaining in ISPA-28 dose response studies for 7G8 and GB4 (blue and red circles, respectively); *n* = 3 trials at each concentration. Solid line represents best fit to *y = a*/(1 + (*x*/*b*)) + (1-*a*)/(1 + (*x*/*c*)).
(PDF)

S2 Fig PCR checks for integration in *C3-TetR*. Ethidium-stained gels using primers with positions indicated in Fig 2A and DNA from the *C3-TetR* transfectant and its KC5 wild-type parent. In each gel, 9 primer pairs that detect homology-directed integration of the pBAC-Dd2C3-TetR-DOZI plasmid are shown, followed by primer pairs specific for the wild-type genomic site (WT) or retention of the episome (Epi). As predicted for recombination, the *C3-TetR* clone yields bands for each integration primer pair and has lost the WT band. KC5 template yields only the WT band. Expected sizes for each primer pair (in kb): *p*2-*p*7, 3.4; *p*1-*p*7, 5.3; *p*2-*p*8, 2.4; *p*1-*p*8, 4.4; *p*2-*p*9, 4.0; *p*5-*p*10, 4.3; *p*4-*p*10, 3.3; *p*5-*p*6, 2.6; *p*4-*p*6, 1.7; *p*2-*p*6, 1.7; *p*4-*p*3, 1.7. Primer sequences are provided in S2 Table.
(TIF)

S3 Fig CLAG3 conditional knockdown in 7G8, a parasite with two *clag3* genes. (A) Schematic showing the 7G8 wild-type locus and the result of homologous recombination of pBAC-*Dd2C3-TetR-DOZI* in the *7G8-TetR* transfection clone. Primer positions are shown with sequences listed in S2 Table. (B) Ethidium-stained gels using primers with positions indicated in S3A Fig and DNA template from *7G8-TetR* and the 7G8 wild-type parent. Integration, wild-type (WT), and episome (Epi) primer pairs are shown with specificity for *clag3*.*1* or *clag3*.*2* indicated in parentheses; a primer pair that yields an integration or wild-type amplicon with either *clag3* gene is indicated as “e” within parentheses. These results reveal integration into *clag3*.*1* with a preserved wild-type *clag3*.*2* gene in *7G8-TetR*. Expected sizes for each primer pair (in kb): *p*2-*p*7, 3.4; *p*12-*p*7, 5.3; *p*1-*p*7, 5.3; *p*2-*p*8, 2.4; *p*12-*p*8, 4.4; *p*1-*p*8, 4.4; *p*2-*p*9, 4.0; *p*4-*p*10, 3.3; *p*4-*p*11, 3.3; *p*5-*p*6, 2.6; *p*4-*p*6, 1.7; *p*2-*p*6, 1.7; *p*4-*p*3, 1.7; *p*2-*p*10, 3.5; *p*2-*p*11, 3.5. Primer sequences are provided in S2 Table. (C) Immunoblots using antibodies against the C-terminus of CLAG3 and the HA epitope tag to probe total cell lysates from indicated parasites cultivated with or without 2 μM aTc. Bottom, Ponceau S staining of hemoglobin as a loading control. (D) Mean ± S.E.M. residual chimeric CLAG3.1_7G8-Dd2_ protein in *7G8-TetR* parasites upon aTc removal, normalized to 100% for matched cultures maintained on aTc (estimated from *n* = 3 independent harvests and anti-HA immunoblots as in panel C). (E) Mean ± S.E.M. normalized expression of the two *clag3* paralogs in *7G8-TetR* cultivated with and without aTc for more than 2 months (black and red bars, respectively; *n* = 3). Note the statistically significant upregulation of the endogenous *clag3*.*2* gene upon conditional knockdown of *clag3*.*1* in this transfectant (asterisk; *P* = 0.02, *n* = 3). Transcription of *clag3*.*1* and the stage-specific *msp2* control were not significantly altered by aTc removal. (F-G) Osmotic lysis kinetics for indicated lines, with or without aTc. Traces reflect lysis kinetics with 0, 0.3, 0.6, 1.8, 5, or 15μM ISPA-28 (top to bottom in each panel, respectively). (H) Dose responses for ISPA-28 inhibition for 7G8 (black circles) and *7G8-TetR* grown with or without aTc (blue and red triangles, respectively). Symbols represent mean ± S.E.M. permeabilities, normalized to 1.0 for matched traces without inhibitor; *n* = 3–7 trials at each concentration. Solid lines represent best fits to *y = a*/(1 + (*x*/*b*)) + (1-*a*)/(1 + (*x*/*c*)). (I) Mean ± S.E.M. block by 15 μM ISPA-28 for indicated parasites and aTc addition, normalized to 100% block for *C3-TetR* cultivated with aTc. aTc removal has a modest effect on channel block despite ~10-fold reduction in expression of the chimeric CLAG3 that encodes ISPA-28 sensitivity.
(TIF)

S4 Fig PCR checks for integration in *C3h-KO* and *7G8-KO* parasites. Ethidium-stained gels using primers with positions indicated in Fig 3A and DNA from indicated knockout parasites, their corresponding parental lines, and the transfection plasmid. Primer pairs specific for integration, the wild type locus, and unintegrated plasmid are indicated in parentheses (*i*, *wt*, or *p*, respectively); in panel B, primer specificity for *clag3*.*1* or *clag3*.2 is also indicated. (A) *C3h-KO* is a clonal knockout that retains the *pL6-c3ko-hdhfr* transfection plasmid. Expected sizes (in kb): *p*13-*p*15, 0.42; *p*17-*p*18, 0.55; *p*13-*p*16, 0.69; *p*17-*p*14, 0.50. (B) *7G8-KO* is a *clag3*.*2* knockout clone with a wild-type *clag3*.*1* locus and retained plasmid. Expected sizes (in kb): *p*12-*p*15, 0.18; *p*13-*p*15, 0.42; *p*17-*p*18, 0.55; *p*12-*p*16, 0.34; *p*13-*p*16, 0.69; *p*17-*p*14, 0.50. Primer sequences are in S2 Table.
(TIF)

S1 Table Annotated genes in the mapped locus. (XLSX)

S2 Table Primers used in this study. (XLSX)
