# Supplementary material for: Complex nutrient channel phenotypes despite Mendelian inheritance in a Plasmodium falciparum genetic cross
Source: PLoS Pathog. 2020 Feb 18;16(2):e1008363. doi: 10.1371/journal.ppat.1008363 (PMC7048409; doi:10.1371/journal.ppat.1008363)

A

```
                930                                                            990
Dd2_3.1     SVNNVFFMNVANNYSKLNTEEREIEIHNSMASRYYAKTMFAAFQMLFSTMLSNNVDNLDKAYGLSENIQV
7G8_3.1     SVNNVFFMNVANNYSKLNKEEREIEIHNSMASRYYAKTMFAAFQMLFSTMLSNNVDNLDKAYGLSENIQV
7G8_3.2     SVNNVFFMNVANNYSKLNKEEREIEIHNSMASRYYAKTMFAAFQMLFSTMLSNNVDNLDKAYGLSENIQV
GB4_3h      SVNNVFFMNVANNYSKLNKEEREIEIHNSMASRYYAKTMFAAFQMLFSTMLSNNVDNLDKAYGLSENIQV
Consensus   SVNNVFFMNVANNYSKLNkEEREIEIHNSMASRYYAKTMFAAFQMLFSTMLSNNVDNLDKAYGLSENIQV

                1000                                                           1060
Dd2_3.1     ATSTSAFLTFAYVYNGSIMDSITNSLLPPYAKKPITQLKYGKTFVFSNYFMLASKMYDMLNYKNLSLLCE
7G8_3.1     ATSTSAFLTFAYVYNGSIMDSVTNSLLPPYAKKPITQLKYGKTFVFSNYFMLASKMYDMLNYKNLSLLCE
7G8_3.2     ATSTSAFLTFAYVYNGSIMDSMTNSLLPPYAKKPITQLKYGKTFVFSNYFMLASKMYDMLNYKNLSLLCE
GB4_3h      ATSTSAFLTFAYVYNGSIMDSVTNSLLPPYAKKPITQLKYGKTFVFSNYFMLASKMYDMLNYKNLSLLCE
Consensus   ATSTSAFLTFAYVYNGSIMDS.TNSLLPPYAKKPITQLKYGKTFVFSNYFMLASKMYDMLNYKNLSLLCE

                1070                                                           1130
Dd2_3.1     YQAVASANFYSAKKVGQFIGRKFLPITTYFLVMRISWTHAITTGQHLIPQLTDPEYGQTPKGKDASGTCP
7G8_3.1     YQAVASANFYSAKKVGQFIGRKFLPITTYFLVMRISWTHAYTTGSHLIAAFD-------PKSSTGTNSQC
7G8_3.2     YQAVASANFYSAKKVGQFIGRKFLPITTYYLVMRISWTHFYITGSHLITYFN-------SSNTDNSSTI-
GB4_3h      YQAVASANFYSAKKVGQFIGRKFLPITTYFLVMRISWTHAFTTGQHLIAAFDPLNTNTSPKPNGGSGI--
Consensus   YQAVASANFYSAKKVGQFIGRKFLPITTYFLVMRISWTHaytTGsHLI..f........pk..d.s.t--
                                                            [         HVR         ]

                1140                                                           1200
Dd2_3.1     SAGLEKCTNYRAPGSFFFTHGLAAEASKYLFFYFFTNLYLDAYKSFPGGFGPAIKEQTQHVQEQTYERKP
7G8_3.1     NGG-----NYKSPESFFFTHGLAAEASKYLFFYFFTNLYLDAYKSFPGGFGPAIKEQTQHVQEQTYERKP
7G8_3.2     ---------NKSPVNFFFTHGLAAEASKYLFFYFFTNLYLDAYKSFPGGFGPAIKEQTQHVQEQTYERKP
GB4_3h      ---------YKSPESFFFTHALAAEASKYLFFYFFTNLYLDAYKSFPGGFGPAIKEQTQHVQEQTYERKP
Consensus   .........yksPesFFFTHgLAAEASKYLFFYFFTNLYLDAYKSFPGGFGPAIKEQTQHVQEQTYERKP
            [   HVR   ]

                1210                                                           1270
Dd2_3.1     SVHSFNRNFFMELANGFMYAFCFFAISQMYAYFENINFYITSNFRFLDRYYGVFNKYFINYARIKLKEIT
7G8_3.1     SVHSFNRNFFMELANGFMYAFCFFAISQMYAYFENINFYITSNFRFLDRYYGVFNKYFINYARIKLKEIT
7G8_3.2     SVHSFNRNFFMELANGFMYAFCFFAISQMYAYFENINFYITSNFRFLDRYYGVFNKYFINYARIKLKEIT
GB4_3h      SVHSFNRNFFMELANGFMYAFCFFAISQMYAYFENINFYITSNFRFLDRYYGVFNKYFINYARIKLKEIT
Consensus   SVHSFNRNFFMELANGFMYAFCFFAISQMYAYFENINFYITSNFRFLDRYYGVFNKYFINYARIKLKEIT

                1280                                                           1340
Dd2_3.1     SDLLIKYEREAYLSMKKYGYLGEVIAARLSPKDKIMNYVHETNEDIMSNLRRYDMENAFKNKMSTYVDDF
7G8_3.1     SDLLIKYEREAYLSMKKYGYLGEVIAARLSPKDKIMNYVHETNEDIMSNLRRYDMENAFKNKMSTYVDDF
7G8_3.2     SDLLIKYEREAYLNMKKYGYLGEVIAARLSPKDKIMNYLHETNDDVMSNLRRYDMENAFKNKMVTYVDDF
GB4_3h      SDLLIKYEREAYLSMKKYGYLGEVIAARLSPKDKIMNYVHETNDDVMSNLRRYDMENAFKNKMSTYVDDF
Consensus   SDLLIKYEREAYLsMKKYGYLGEVIAARLSPKDKIMNYvHETNeDiMSNLRRYDMENAFKNKMsTYVDDF
                           [                    CLAG3 antibody                   ]
```

```
            1350                                                        1410
Dd2_3.1     AFFDDCGKNEQFLNERCDYCPVIEEVEETQLFTTTGDKNTNKTTEIKKQTSTYIDTEKMNEADSADSDDE
7G8_3.1     AFFDDCGKNEQFLNERCDYCPVIEEVEETQLFTTTGDKNTNKTTEIKKQTSTYIDTEKMNEADSADSDDE
7G8_3.2     AFFDDCGKNEQFLNERCDYCPVIEEVEETELFTTTGDKNTNETTEIKKQTSTYIDTEKMNEADSADSDDE
GB4_3h      AFFDDCGKNEQFLNERCDYCPVIEEVEETELFTTTGDKNTNKTTEIKKQTSTYIDTEKMNEADSADSDDE
Consensus   AFFDDCGKNEQFLNERCDYCPVIEEVEETqLFTTTGDKNTNkTTEIKKQTSTYIDTEKMNEADSADSDDE
            [CLAG3 antibody]

            1420      1430
Dd2_3.1     KDSDTPDDELMISRFH
7G8_3.1     KDSDTPDDELMISRFH
7G8_3.2     KDFDTPDNELMIARFH
GB4_3h      KDSDTPDNELMIARFH
Consensus   KDsDTPDdELMIaRFH
            [CLAG3 antibody]
```

B

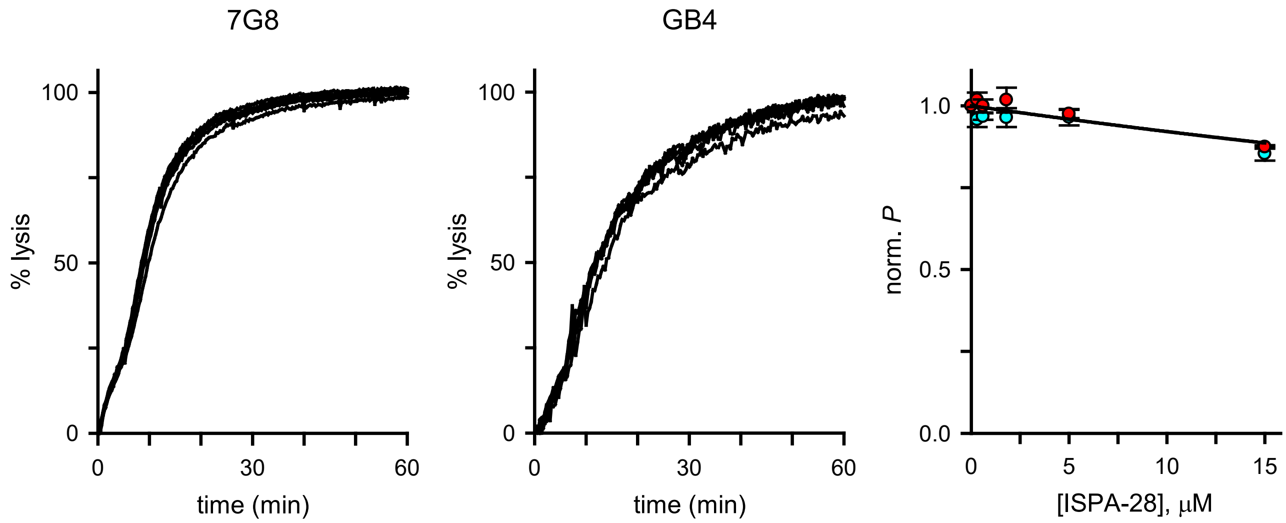

Supplement: S1 Fig — (A) Multiple sequence alignment of indicated CLAG3 sequences from Dd2, 7G8, and GB4 lines. The C-terminal fragment expressed by the last exon of clag3 is shown; CLAG3 sequences upstream of this region are also highly conserved. A single hypervariable region (HVR) in CLAG3 proteins and the region used for production of an anti-CLAG3 polyclonal antibody are both labeled. (B) Sorbitol-induced osmotic lysis kinetics for 7G8 and GB4 lines with 0, 0.3, 0.6, 1.8, 5, or 15μM ISPA-28 (top to bottom, respectively in each panel). Right panel shows mean ± S.E.M. permeability remaining in ISPA-28 dose response studies for 7G8 and GB4 (blue and red circles, respectively); n = 3 trials at each concentration. Solid line represents best fit to y = a/(1 + (x/b)) + (1-a)/(1 + (x/c)). (PDF) [file ppat.1008363.s001.pdf]
