# Supplementary figures and images for: Complex nutrient channel phenotypes despite Mendelian inheritance in a Plasmodium falciparum genetic cross
Source: PLoS Pathog. 2020 Feb 18;16(2):e1008363. doi: 10.1371/journal.ppat.1008363 (PMC7048409; doi:10.1371/journal.ppat.1008363)

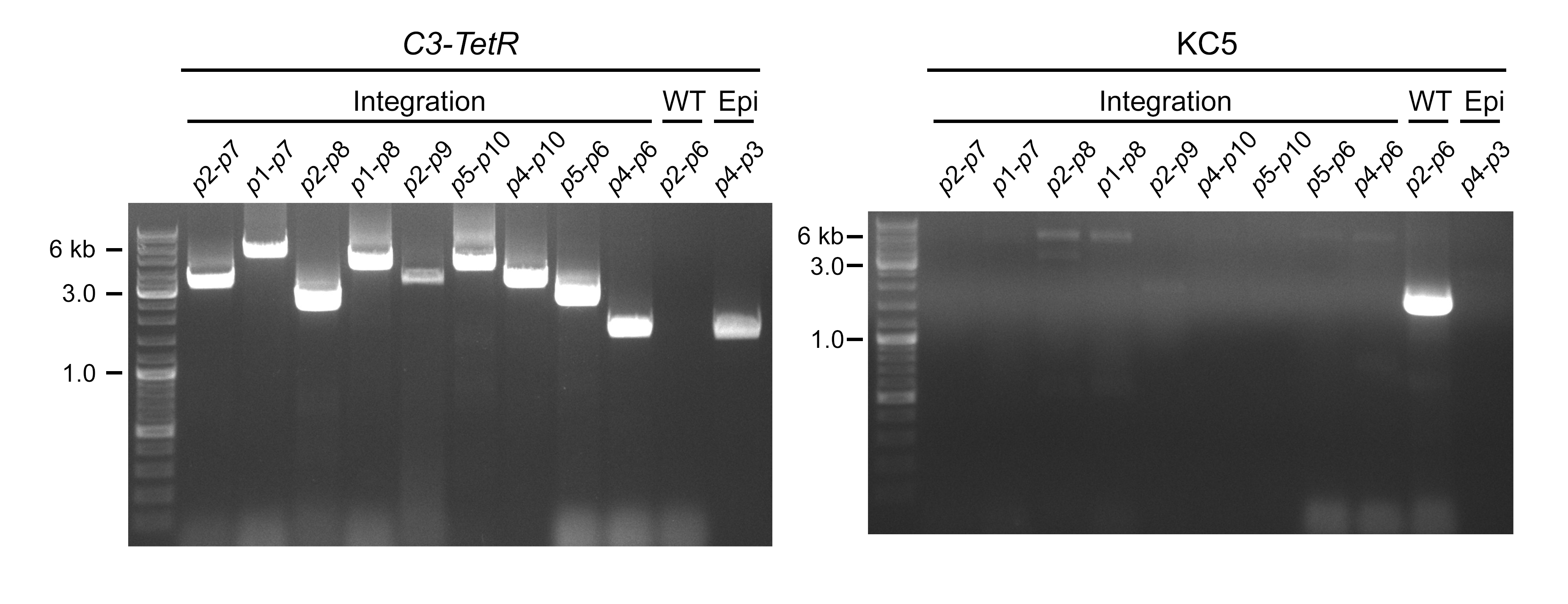

Supplement: S2 Fig — Ethidium-stained gels using primers with positions indicated in Fig 2A and DNA from the C3-TetR transfectant and its KC5 wild-type parent. In each gel, 9 primer pairs that detect homology-directed integration of the pBAC-Dd2C3-TetR-DOZI plasmid are shown, followed by primer pairs specific for the wild-type genomic site (WT) or retention of the episome (Epi). As predicted for recombination, the C3-TetR clone yields bands for each integration primer pair and has lost the WT band. KC5 template yields only the WT band. Expected sizes for each primer pair (in kb): p2-p7, 3.4; p1-p7, 5.3; p2-p8, 2.4; p1-p8, 4.4; p2-p9, 4.0; p5-p10, 4.3; p4-p10, 3.3; p5-p6, 2.6; p4-p6, 1.7; p2-p6, 1.7; p4-p3, 1.7. Primer sequences are provided in S2 Table. (TIF) [file ppat.1008363.s002.tif]

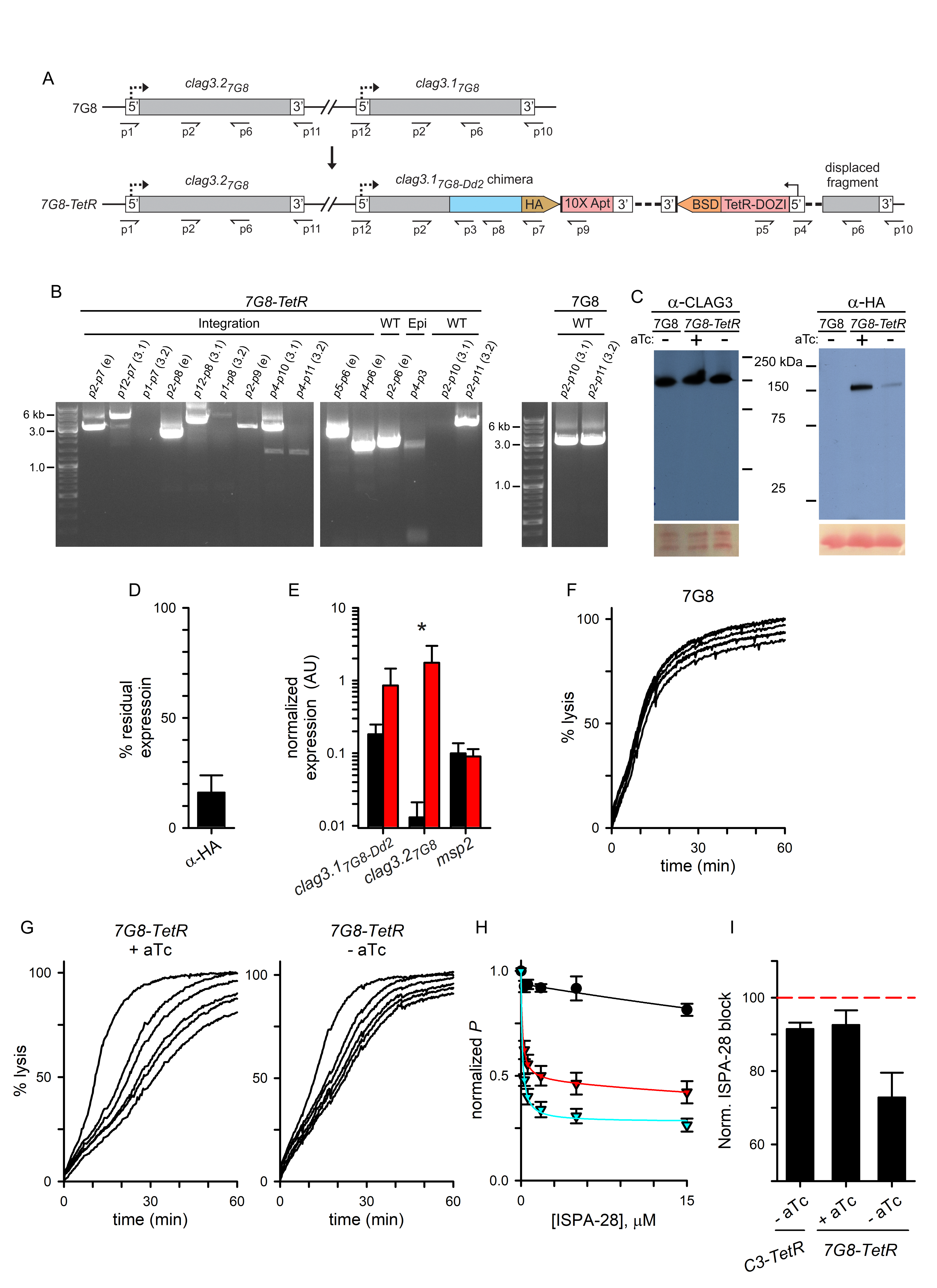

Supplement: S3 Fig — (A) Schematic showing the 7G8 wild-type locus and the result of homologous recombination of pBAC-Dd2C3-TetR-DOZI in the 7G8-TetR transfection clone. Primer positions are shown with sequences listed in S2 Table. (B) Ethidium-stained gels using primers with positions indicated in S3A Fig and DNA template from 7G8-TetR and the 7G8 wild-type parent. Integration, wild-type (WT), and episome (Epi) primer pairs are shown with specificity for clag3.1 or clag3.2 indicated in parentheses; a primer pair that yields an integration or wild-type amplicon with either clag3 gene is indicated as “e” within parentheses. These results reveal integration into clag3.1 with a preserved wild-type clag3.2 gene in 7G8-TetR. Expected sizes for each primer pair (in kb): p2-p7, 3.4; p12-p7, 5.3; p1-p7, 5.3; p2-p8, 2.4; p12-p8, 4.4; p1-p8, 4.4; p2-p9, 4.0; p4-p10, 3.3; p4-p11, 3.3; p5-p6, 2.6; p4-p6, 1.7; p2-p6, 1.7; p4-p3, 1.7; p2-p10, 3.5; p2-p11, 3.5. Primer sequences are provided in S2 Table. (C) Immunoblots using antibodies against the C-terminus of CLAG3 and the HA epitope tag to probe total cell lysates from indicated parasites cultivated with or without 2 μM aTc. Bottom, Ponceau S staining of hemoglobin as a loading control. (D) Mean ± S.E.M. residual chimeric CLAG3.17G8-Dd2 protein in 7G8-TetR parasites upon aTc removal, normalized to 100% for matched cultures maintained on aTc (estimated from n = 3 independent harvests and anti-HA immunoblots as in panel C). (E) Mean ± S.E.M. normalized expression of the two clag3 paralogs in 7G8-TetR cultivated with and without aTc for more than 2 months (black and red bars, respectively; n = 3). Note the statistically significant upregulation of the endogenous clag3.2 gene upon conditional knockdown of clag3.1 in this transfectant (asterisk; P = 0.02, n = 3). Transcription of clag3.1 and the stage-specific msp2 control were not significantly altered by aTc removal. (F-G) Osmotic lysis kinetics for indicated lines, with or without aTc. Traces reflect [file ppat.1008363.s003.tif]

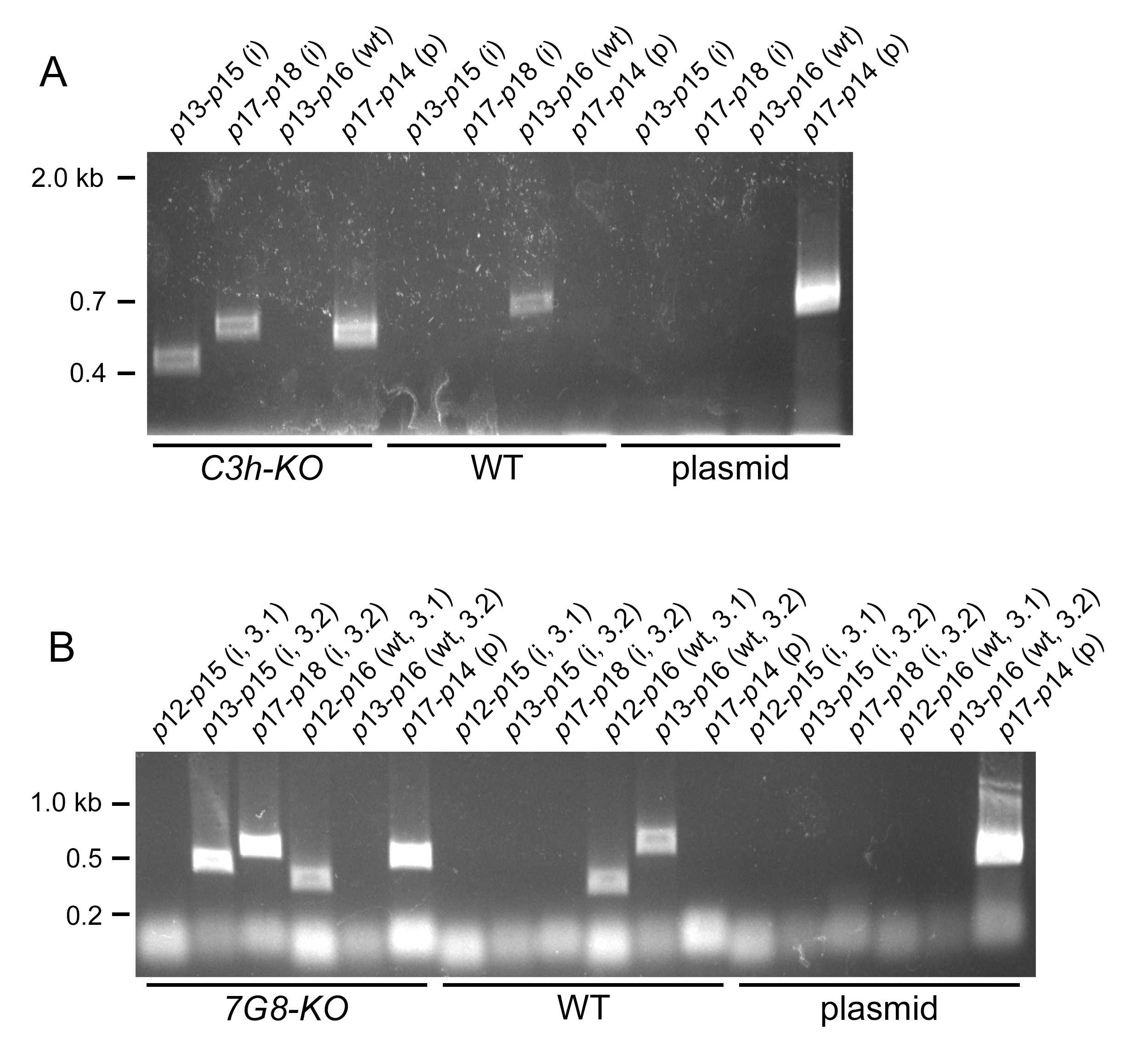

Supplement: S4 Fig — Ethidium-stained gels using primers with positions indicated in Fig 3A and DNA from indicated knockout parasites, their corresponding parental lines, and the transfection plasmid. Primer pairs specific for integration, the wild type locus, and unintegrated plasmid are indicated in parentheses (i, wt, or p, respectively); in panel B, primer specificity for clag3.1 or clag3.2 is also indicated. (A) C3h-KO is a clonal knockout that retains the pL6-c3ko-hdhfr transfection plasmid. Expected sizes (in kb): p13-p15, 0.42; p17-p18, 0.55; p13-p16, 0.69; p17-p14, 0.50. (B) 7G8-KO is a clag3.2 knockout clone with a wild-type clag3.1 locus and retained plasmid. Expected sizes (in kb): p12-p15, 0.18; p13-p15, 0.42; p17-p18, 0.55; p12-p16, 0.34; p13-p16, 0.69; p17-p14, 0.50. Primer sequences are in S2 Table. (TIF) [file ppat.1008363.s004.tif]
